# Studying the Stability of Anthocyanin Pigments Isolated from Juices of Colored-Fleshed Potatoes

**DOI:** 10.3390/ijms252011116

**Published:** 2024-10-16

**Authors:** Agnieszka Tkaczyńska, Esther Sendra, Nuria Jiménez-Redondo, Elżbieta Rytel

**Affiliations:** 1 Department of Food Storage and Technology, Wrocław University of Environmental and Life Sciences, 37 Chełmońskiego Str., 51-630 Wrocław, Poland; 2 Institute on Agrofood and Agroenvironmental Research and Innovation (CIAGRO-UMH), Miguel Hernandez University of Elche, Carretera de Beniel, km 3.2, 03312 Orihuela, Spain; esther.sendra@umh.es (E.S.); n.jimenez@umh.es (N.J.-R.)

**Keywords:** potato juices, fruit and vegetable concentrates, pasteurization, color stability, anthocyanins

## Abstract

The aim of this study was to obtain extracts of anthocyanin pigments from red and purple-fleshed potato juices characterized by stable color. For this purpose, potato juices were pasteurized at different temperatures or fruit and vegetable concentrates were added to them. Color stability tests of the obtained pigments were carried out in model pH and temperature conditions and after adding to natural yogurt. Both the pasteurization process and the addition of fruit and vegetable concentrates to the potato juices positively affected their color and its stability in time. However, the pasteurization of the potato juices had a negative effect on the content of biologically active compounds, in contrast to the juices stabilized with the addition of fruit and vegetable concentrates. Anthocyanin pigments from red-fleshed potato juices were more stable than those isolated from the purple-fleshed potato juices. The results of model tests of the anthocyanin pigment concentrates from the colored-flesh potatoes and natural yoghurts with their addition confirmed the high stability of the tested concentrates.

## 1. Introduction

Anthocyanins belong to the group of polyphenolic compounds (flavonoids) and represent plant pigments classified as natural non-nutritive substances. They are highly diversified in terms of structure, as well as chemical, biological and physical properties [1,2]. In plants, they occur in the form of granules of various sizes in vacuoles of mainly fruits, stems, flowers, and leaves, endowing them with colors from yellow through orange and different hues of red, purple, and blue to black [1]. Anthocyanins are water-soluble glycosides and acylglycosides, which are broken down upon acidic hydrolysis to saccharides and colored aglycones, i.e., anthocyanidins. In plant raw materials, anthocyanidins occur in the form of polyhydroxylated and polymethoxylated heterosides derived from flavylium or 2-phenylbenzopyrrolic ions [2]. Several hundred anthocyanins have been identified so far, differing in anthocyanidin type, as well as in the number and site of attachment of a sugar residue and its substituents. Six anthocyanidins have been found to occur in fruit and vegetables: cyanidin, delphinidin, pelargonidin, peonidin, malvidin, and petunidin [3]. The color of anthocyanins is determined not only by their chemical structure but also by environment (pH, contents of sugars and their degradation products, SO_2_, and the presence of co-pigments), or external conditions (temperature, light, access of oxygen) [4]. Fruit and vegetables differ in the type and structure of anthocyanins. According to several authors [2,5], anthocyanins derived from vegetables are more stable than those found in fruits, which is chiefly, due to their structure, which was found to be positively affected by glycosylation at positions 3 and 5 and the presence of acyl groups. Furthermore, anthocyanins possessing one or two aromatic acyl groups undergo intramolecular co-pigmentation which also improves their stability [5,6,7]. Bearing in mind the growing demands of contemporary consumers, food producers increasingly often make use of natural food additives [5,8]. However, pigments derived from natural sources may be successfully replaced by their synthetic counterparts, which are used, e.g., for coloring bakery products, pastry and confectionery products, meat products, and beverages [4,5,9]. The most common pigments used worldwide in this respect are those derived from grapes and grape pomace [10]. However, in some European countries, food producers use also anthocyanins isolated from blackcurrants, chokeberries and blackberries [11]. Apart from their attractive color, anthocyanins also exhibit anti-inflammatory, antioxidative, and anticarcinogenic properties, which have been confirmed in both in vivo and in vitro studies [12,13]. Despite their multiple advantages, anthocyanins are, however, little resistant to processes applied in the fruit and vegetable processing industry, which diminishes their applicability as food additives [14]. The major challenge faced by food producers is to ensure the stable color of these compounds and products with their addition [3,15], and color is one of the major quality attributes of food affecting its acceptability by consumers. This problem can be solved by producing anthocyanin pigments from raw materials containing acylated anthocyanins [3], like, e.g., potatoes of red-fleshed and purple-fleshed cultivars [16,17]. Potatoes are widely available in many countries, easy to grow, and generally cheaper than fruits [18]. Currently, the market offers pigments isolated from red cabbage [19], while there are no anthocyanin pigments derived from potatoes [5]. Another means to improve the color stability of anthocyanins is to lower environment pH because acidic solutions are the perfect media for the co-pigmentation phenomenon to occur and for the prevalence of the flavylium cation imparting orange, red or purple colors [1]. Another factor likely to improve color stability of anthocyanins is the pasteurization process. The effect of high temperature on the raw material causes the inactivation of enzymes involved in the enzymatic darkening processes of products. However, it should be noted that too high a temperature reduces color intensity, shifting the balance between anthocyanins towards a colorless carbinol base and chalcone forms [3,6,14,20,21]. The mechanism of anthocyanin degradation is not fully understood and explained in the scientific literature. One of the possible factors influencing the decrease in their stability is their reaction with quinones, compounds with strong oxidizing properties, which are formed as a result of the enzymatic oxidation of phenolic compounds, mainly chlorogenic acid and catechins. Phenolic compounds, primarily chlorogenic acid, caffeic acid, and catechins, are potential substrates for phenoloxidases—enzymes involved in enzymatic darkening processes occurring in processed fruits and vegetables. As a result of this process, polyphenols are oxidized to quinones, which results in darkening of the color of products (juices) and, consequently, a decrease in their quality. Quinones formed during the enzymatic oxidation of polyphenols can react with anthocyanins, causing their oxidative polymerization [22,23]. Studies on factors influencing the color and anthocyanin composition of juices obtained from colored-fleshed potatoes are still poorly described in the scientific literature. Potato juice may become a good source of color-stable anthocyanin pigments in the future.

The aim of this study was to obtain anthocyanin pigments isolated from red- and purple-fleshed potato juices characterized by stable color. For this purpose, potato juices were pasteurized at different temperatures or fruit and vegetable concentrates were added to them. The color stability studies of the obtained pigments were carried out under model pH and temperature conditions and after the addition to natural yogurt.

## 2. Results and Discussion

### 2.1. Characterization of Potato Juices

Table 1 presents data demonstrating color changes observed in the juices prepared from the red-fleshed and purple-fleshed potato cultivars. When analyzed immediately after preparation, the control samples of juices obtained from the purple-fleshed potato tubers (CSP) had a darker color (L* = 0.43), a smaller contribution of red color (a* = 1.77), and a greater contribution of blue color (b* = 0.45) in the color profile compared to the control juice samples made of red-fleshed potatoes (CSR) (L* = 0.56, a* = 3.06, b* = 0.92). Tkaczyńska and Rytel [18] and Iborra-Bernarda et al. [24] also observed a darker color in purple-fleshed potato tubers. The intensity of the color of colored-fleshed potato tubers depends primarily on the composition and content of their anthocyanins [3]. Both pasteurization and the addition of juice concentrates were found to positively affect the color of the produced juices (Table 1). In addition, the pasteurized juices had a lighter color (mean L* = 18.13) and a greater contribution of red color (mean a* = 27.10) and yellow color (b* = 22.64) in the color profile compared to the juices with concentrate addition (L* = 3.83, a* = 12.31, b* = 4.81). Tkaczyńska et al. [3,25] also observed a positive effect of pasteurization and acidic pH on the color of potato juices. In contrast, Rios-Romero et al. [26] demonstrated color deterioration in potato juices produced from orange-fleshed sweet potato cultivars during heat treatment. Other authors [6,20], who analyzed the color of fruit juices, also reported a negative impact of pasteurization on color hue and stability. In turn, some other authors [2,27] emphasized the positive influence of the acidic environment on color stability in fruit and vegetable juices. According to the authors of [22,23], at low pH (between 1–3), anthocyanins occur in the form of a red flavylium cation, which is the most stable form of these compounds. In addition, during the enzymatic or non-enzymatic browning processes of juices, quinones are formed, which can react with anthocyanins, causing their oxidative polymerization. On the other hand, the presence of organic acids, especially ascorbic acid, which occurs naturally in fruit and vegetable juices and concentrates, can inhibit this process. Because organic acids, thanks to their antioxidant properties, can reduce quinones to phenols.

The results of the analyses conducted in the present study demonstrate changes in the color of potato juices observed 4 h after their preparation (Table 1). After this time, the color of the control samples (CSP, CSR) was darker (the value of the L* parameter decreased by 0.19, on average). A reduction was also noted in the contribution of red color (the a* value decreased by 1.0, on average) and yellow color (the b* value decreased by 0.44, on average). Similar changes in color parameters were noted after 4 h in the pasteurized juices. In turn, the juices with the addition of concentrates analyzed 4 h after their preparation had a lighter color (L* value increase) and a greater contribution of red color (a* value increase) and yellow color (b* value increase) in their color profile (Table 1). The addition of the lime concentrate had the most positive impact on the color of the potato juices produced from the purple-fleshed cultivars, whereas the addition of the rhubarb concentrate had the most positive impact on those made of the red-fleshed tubers. 

Aadil et al. [6] and Rios-Romero et al. [26] also noted a reduction in the values of all the analyzed color parameters of the pasteurized potato juices over time, whereas Tkaczyńska et al. [25] demonstrated a positive effect of fruit and vegetable juice concentrates after 4 h. Temperature increase or pH decrease trigger a series of chemical reactions affecting the color and stability of anthocyanins. Products containing anthocyanins are susceptible to color changes during processing and storage. These changes are largely determined by the type of raw materials, the chemical structure of the anthocyanins found in these materials, and the method of food preservation [28]. Both the color and the stability of anthocyanin compounds are strongly dependent on the substitution of colored aglycones and their form (acylated or non-acylated). Hydroxyl substitutions that are increased in the B ring under the influence of temperature or acidic environment cause a shift in the absorption maximum of the visible light (λmax) towards longer waves, triggering the bathochromic shift, which, in turn, results in a more intense red color in anthocyanins [5,7,29].

Pasteurization temperature and the addition of fruit and vegetable concentrates were found to affect the content of total polyphenols (TPs) (mg GAE/g DM) in juices (Table 2). The pasteurized potato juices had, on average, 12% fewer TPs compared to the control samples (CSs). The highest TPs content determined after pasteurization at a temperature of 65 °C was determined in the juices produced from the red-fleshed Lily Rose cultivar (Table 2). In turn, the addition of juice concentrates to potato juices caused, on average, a 33% increase in their TP content compared to the CSs (Table 2). This could have been due to the total polyphenols content in the fruit and vegetable concentrates (Supplementary Materials Table S1). The highest amount of total polyphenols and the highest antioxidant activity were found in the rhubarb concentrate (Supplementary Materials Table S1). Regarding additions to the potato juices, the highest TPs content determined in the juices was found with the addition of lemon and lime concentrates (Table 2). Both the pasteurization process and the addition of concentrates to the potato juices also affected the composition and content of their total anthocyanins (TAs) (mg/100 g DM) (Table 2, Table 3 and Table 4). However, the temperatures used during the pasteurization process had an adverse effect on the amount and composition of anthocyanins (Table 3). The decrease in the amount of these anthocyanins also affected the color of the juices. Because they are responsible for individual shades of color, e.g., delphinidin gives a blue–violet color, pelargonidin is responsible for an orange–red color, petunidin and malvidin produce purple, and peonidin produces purple–pink [2,3,7,30]. As a result, the color of the juices after pasteurization was lighter (Table 1). The juices produced from the purple-fleshed cultivars had higher contents of petunidin and peonidin, whereas those made from the red-fleshed potato tubers had higher contents of pelargonidin. Pasteurization temperature contributed to TA decrease by 40% on average, compared to the CSs. The highest TAs content was determined in the juices produced from the purple-fleshed Double Fun cultivar and pasteurized at 75 °C (Table 2). The juices with the addition of the fruit and vegetable concentrates had, on average, 52% higher TAs contents compared to the CSs, with the highest TAs content determined in the juices made of the red-fleshed Lily Rose potatoes with the addition of the lemon and lime concentrates.

The lower content of polyphenolic compounds noted in the potato juices after pasteurization may be indicative of their partial degradation [31]. Heating causes anthocyanins to degrade in the course of chemical reactions, such as polymerization or oxidative degradation [32]. The extent of the degradation of anthocyanin pigments depends on their chemical structure. Anthocyanins may appear brighter and less stable when heated because the balance between anthocyanin molecules shifts toward the colorless carbinol base and chalcone forms [33]. Volden et al. [34] confirmed that heat treatment (blanching, cooking, and steam-cooking) reduced the content of anthocyanins by 43%, on average. The losses of polyphenolic compounds in potato or fruit juices induced by high-temperature treatments were also reported by Tkaczyńska et al. [3] and Dobson et al. [32]. Undesirable chemical reactions and the degradation of anthocyanins observed during food processing and storage may be mitigated by reducing the pH of raw materials or the products obtained from them [25]. The pH value of most food products ranges from 3.5 to 7, which has a significant impact on the color and stability of pigments [1]. A change in the pH value to more acidic (between 1 and 3) through the addition of, e.g., organic acids that possess free pairs of electrons, triggers intermolecular co-pigmentation with anthocyanins, making them more stable. A flavylium cation is a key player in this reaction, which imparts intense orange, red, or purple colors [25,35,36]. Many authors [27,28,31,37,38] have reported that the heat treatment of fruit and vegetables at reduced pH elicited desirable effects, manifested by the stabilization and preservation of anthocyanins.

### 2.2. Characterization of Potato Pigments

Both pasteurization and the addition of fruit and vegetable concentrates to potato juices affected the color of the anthocyanin pigments isolated from them (Table 5). The color lightness (L* color parameter) of the isolated pigments ranged from 32.7 (Provita potato juice after pasteurization at 65 °C) to 41.1 (Lily Rose potato juice with the addition of rhubarb) (Table 5). The anthocyanin pigments isolated from the potato juices produced from the purple-fleshed cultivars had a lesser contribution of red color (mean a* = 0.48) and a greater contribution of yellow color (mean b* = −1.01) in the color profile, compared to CSP (a* = 1.40, b* = −2.43) (Table 5). In turn, the juices obtained from the red-fleshed potato tubers had a greater contribution of both red (mean a* = 7.11) and yellow (mean b* = 0.40) in the color profile, compared to CSR (a* = 4.18, b* = −0.31) (Table 5). All the analyzed lyophilized pigments were more stable over time than the juices they were extracted from and had a lighter color (L* value higher by 26.6 on average), a lesser contribution of red color (a* value lower by 12.6 on average), and a greater contribution of blue color (b* value higher by 9.4 on average) (Table 5).

Pasteurization temperature and the addition of fruit and vegetable concentrates were found to positively affect the TPs content (mg GAE/g DM) and also the TAs composition and content (mg/100 g DM) in the juices after their purification (i.e., in lyophilized pigments) (Table 2, Table 6 and Table 7). The pigments had, on average, 10–20 times more TPs and 50–80 times more TAs than the non-purified juices. The addition of fruit and vegetable concentrates decreased the pH of the potato juices, which contributed to the increased stability of their anthocyanins and enabled their even more effective isolation compared to the pasteurized samples. The lyophilized pigments obtained after the purification of the juices with the addition of fruit and vegetable juice concentrates had 1–3-fold higher contents of TPs and TAs than the pasteurized samples (Table 2). It was also found that the stability of lyophilized anthocyanin pigments was most strongly affected by the addition of concentrates from the lemon and rhubarb juices (Table 2). Molecules of anthocyanins found in potatoes and potato juices contain acyl groups, which may positively influence their stability [33,37,38]. In addition, anthocyanins can form complexes with, among others, amino acids, organic acids, flavonoids, metals, nucleotides, alkaloids, and polysaccharides [37]. However, complexes with other compounds may deteriorate their color and reduce their stability, or they may result in the formation of co-pigments featuring a more stable color and structure [2,37,38].

### 2.3. Model Study

The stability of the anthocyanin pigment extracts was also analyzed under model study conditions. It was found that the pasteurization temperatures from 60 °C to 100 °C significantly affect the color (Table 8 and Table S2) and activity of the anthocyanin pigment extracts (Tables S4–S7), and also the content of total polyphenols (Table 9 and Table S3). Color changes were observed along with pasteurization temperature increase, namely the color of the anthocyanins isolated from the purple-fleshed tubers turned brighter (the L* value increased by 35.4, on average), the contribution of red in the color profile decreased (the a* value decreased by 9.20, on average), and so did the contribution of blue color (the b* value decreased by 30.0, on average), compared to the control sample (mean L* = 9.78, mean a* = 22.5, and mean b* = −5.52). In turn, the pigments isolated from the juices made from the red-fleshed potato cultivar were darker (the L* value decreased by 5.8, on average), and had a greater contribution of red (the a* value increased by 3.0, on average) and yellow (the b* value increased by 3.1, on average) in the color profile, compared to the control samples (mean L* = 69.1, a* = 11.9, b* = 27.2) (Table 8).

The conducted analyses demonstrated greater stability in the anthocyanins from the red-fleshed potatoes and lesser changes in the values of their color coordinates compared to the pigments derived from the purple-fleshed potato tubers. The analyzed treatment temperatures also caused different changes in TPs content of the juices after their purification (in the pigment extracts). A lower TPs content (by 7.20 mg GAE/g DM on average) was determined in the pigments obtained from the juices produced from the purple-fleshed potatoes treated at temperatures ranging from 60 °C to 100 °C. In turn, the TPs content increased by 8.43 mg GAE/g DM, on average, in the red pigments, compared to the non-heated ones (Table 9).

Analyses were also conducted to determine the impact of pH on changes in the color (Table 10) and total polyphenol content of the anthocyanin pigment extracts (Table 11). The pigments isolated from the potato juices produced from the purple-fleshed cultivars exhibited the highest levels of antioxidative activity (Table S7) and the highest TPs content at pH 3, and those obtained from the juices made from the red-fleshed tubers showed the highest TPs content at pH 5 (Table 11). The addition of the fruit and vegetable concentrates to the potato juices positively affected the stability of the anthocyanin pigment extracts and caused lesser changes in color (Table 8 and Table 10), in total polyphenols content (Table 9 and Table 11), and in antioxidative activity (Table S7), compared to the control samples and pasteurized samples. The most stable turned out to be the pigments obtained from the juices with rhubarb concentrate addition. In turn, the greatest changes in color antioxidative activity and total polyphenol content were noted in the pigments treated at a temperature of 100 °C at pH 1 and pH 11 (Table 8, Table 9, Table 10, Table 11 and Tables S3–S7). In most of the analyzed pigments, the total polyphenol content was found to increase. Other authors [39,40] demonstrated the degradation of most anthocyanins at pH 7. In turn, Walkowiak-Tomczak and Czapski [41] and Jing et al. [31] demonstrated the enhanced degradation of anthocyanins along with environment pH increase during heat treatment. Reyes and Cisneros-Zevallos [40], who analyzed the thermal stability of anthocyanin solutions prepared from purple-fleshed and red-fleshed sweet potatoes, showed the higher stability of these pigments under conditions of low pH and temperature. Furthermore, Fan et al. [42], who investigated the color stability of anthocyanins from purple-fleshed sweet potatoes fermented at pH 2–7, demonstrated that they were more stable in a strongly acidic environment (pH 2.0–4.0) than in a slightly acidic one (pH 5.0–6.0). In turn. Li et al. [27] analyzed the effect of heat treatment at 80, 90, and 100 °C on the stability of anthocyanin pigments obtained from purple-fleshed potatoes with the addition of fruit juices with pHs from 2 to 6 and observed their greater stability at pH 3 and pH 4 during heat treatment in the analyzed range of temperatures. i.e., 80–100 °C.

### 2.4. Analysis of Natural Yogurt with Added Anthocyanin Pigments

All the analyzed yoghurts with the addition of lyophilized pigments had darker color (lower L* values) and a greater contribution of red color in the color profile (higher a* values) that the natural yoghurt (Table 12). The color parameters of the analyzed yoghurts were stable and remained unchanged after 7 days of storage (Table 12). According to García-Perez et al. [43], the L*, a*, and b* color parameters of cold-stored yoghurts are stable. The color of natural yoghurts is mainly affected by the pH and the additives used, like, e.g., dietary fiber.

The yoghurts with the addition of lyophilized pigments from juices with fruit and vegetable concentrates analyzed immediately after preparation had a higher pH than the natural yoghurt (Table 13). In turn, the pH of the yoghurts with the addition of pigments obtained from the pasteurized juices approximated that of the natural yoghurt or were slightly lower, in the range of 3.92 to 4.02 (Table 13). After 7 days of storage, the pH values of all the yoghurts increased, with the highest value noted for the natural yoghurt (pH = 4.15) and for the yoghurt with the addition of red pigments—the control sample (without the addition of fruit and vegetable concentrates and non-pasteurized) (Table 13).

It was also found that most of the yoghurts with lyophilized pigments from the pasteurized juices had less lactic acid bacteria compared to the natural yoghurt and to the yoghurts with pigments from the juices with fruit and vegetable concentrate addition. The highest counts of Lab-MRS-5 and Lac-MS17-7 strain bacilli were determined in the yoghurts with pigments from the juices with rhubarb concentrate addition (Table 13). An increase in pH in the yoghurts noted after their 7-day storage caused a decrease in the total numbers of Lab-MRS-5 and Lac-MS17-7 in most of the analyzed yoghurts, except for the samples with the addition of pigments from the red-fleshed potato juices pasteurized at 65 °C and the lemon concentrate, as well as those with the pigments from the control purple-fleshed potato juices and from the purple-fleshed potato juices pasteurized at 75 °C (Table 13). According to Sendra et al. [44], probiotic bacteria population decreases and the acidity of dairy beverages increases (pH drops) with the extension of their storage time. Similar observations were made by Szołtysik et al. [45], who analyzed yoghurts with the addition of dried extracts from blue honeysuckle berries.

**Figure 1:**
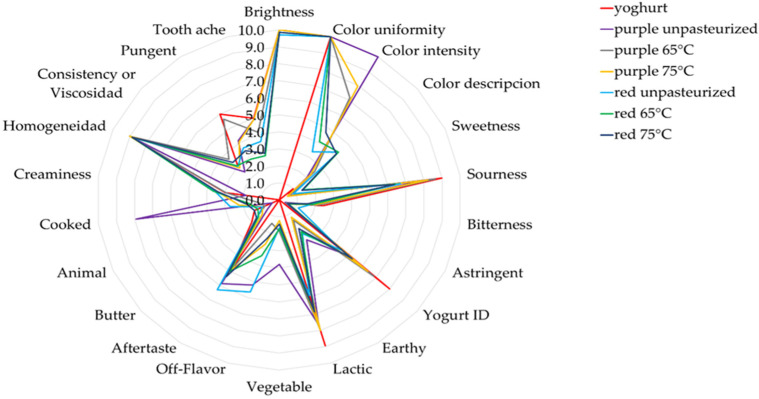
Sensory evaluation of yogurts without (natural yogurt) and with added pigments obtained from pasteurized and unpasteurized juices. 0—lowest score; 10—highest score.

**Figure 2:**
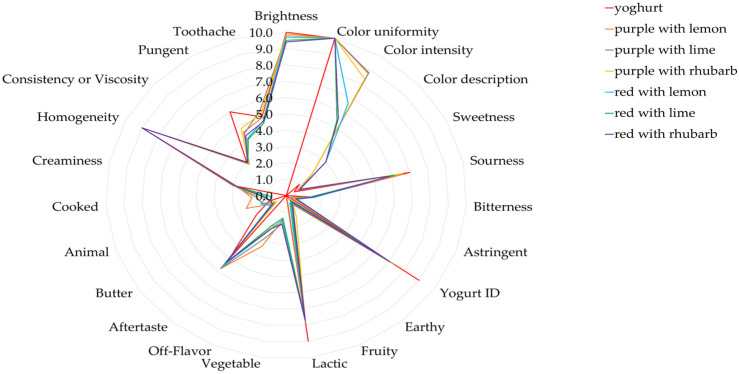
Sensory evaluation of yogurts without (natural yogurt) and with added pigments obtained from juices with added fruit and vegetable concentrates. 0—lowest score; 10—highest score.

The yoghurts with the addition of pigments received different scores to the natural yoghurt in the organoleptic assessment of their taste, color, aroma, and consistency (Figure 1 and Figure 2). Differences were demonstrated in the color assessment, i.e., the yoghurts with lyophilized pigments from juices with the addition of the fruit and vegetable juice concentrates were assessed as darker and as having a more intense color compared to the yoghurts with pigments from the pasteurized juices and the natural yoghurt (Figure 1 and Figure 2). All the yoghurts with pigments added were evaluated as less sour, which was also confirmed by their pH measurements (Table 12, Figure 2). In turn, the taste of the analyzed yoghurts with lyophilized pigments from the juices with the addition of fruit and vegetable juice concentrates were typical. i.e., buttery and sour (Figure 1). The taste of the yogurts with added colorants obtained from pasteurized juices was pungent and cooked, had a strange aftertaste and a sharp sourness, and was not acceptable (Figure 2).

## 3. Materials and Methods

### 3.1. Colored-Fleshed Potato Juices

The experimental materials were potatoes from which juices and anthocyanin pigment were obtained. The potato juices were produced from tubers of one red-fleshed potato cultivar, Lily Rose, and two purple-fleshed cultivars, Provita and Double Fun. The potatoes were sourced directly from Polish producers from the growing season 2021–2022. Potatoes for the study were harvested at full physiological maturity in September–October. The raw materials were stored for 1–4 weeks in a temperature-controlled storage room until potato juices were obtained from it. Lemon, lime, and rhubarb juice concentrates used in this study were purchased from Döhler Holland B.V., Oosterhout, The Netherlands.

#### 3.1.1. Preparation of Potato Juices

Potato juices were produced from ca. 30 kg portions of potatoes randomly selected from each cultivar that were washed and dried. The juice was extracted from non-peeled potatoes using an automatic juice extractor, Robot Coupe J100 (Machej Holding Sp. z o.o., Gliwice, Poland). The juices obtained were divided into six portions, each of ca. 1.5 L. The first portion was non-pasteurized juice without the addition of the fruit and vegetable juice concentrates (control sample). The remaining samples of juices were supplemented with the concentrates and pasteurized.

##### Potato Juices with the Addition of Fruit and Vegetable Juice Concentrates

Aqueous solutions of lemon, lime, and rhubarb juice concentrates were added directly to potato juices during their preparation. The concentrates were added in doses of 1% to the juices made from the purple-fleshed potato tubers and of 2% to those made of the red-fleshed potatoes. The juices with the concentrates (47% Brix) were left in a dark room for 45 min to enable starch separation via sedimentation. Subsequently, they were filtered through a filtration cloth and centrifuged using an MPW−351R centrifuge at 1000 rpm and temperature of 9 °C, for 10 min, to achieve clear juices.

##### Pasteurized Juices

Some of the clear potato juices without the concentrates (see Section 3.1.1) were pasteurized at a temperature of 65 °C for 5 min or at a temperature of 75 °C for 5 min. After pasteurization, the juices were cooled and centrifuged on an MPW-351R centrifuge at 3000 rpm, at temperature of 9 °C, for 5 min to obtain clear solutions. Non-pasteurized juice without the addition of fruit and vegetable juice concentrates served as the control sample.

#### 3.1.2. Preparation of Potato Pigments

Anthocyanin pigments were isolated from non-pasteurized and pasteurized potato juices with and without the addition of fruit and vegetable juice concentrates via gel chromatography. A chromatographic column was filled with “Amberlite XAD 16” resin. Potato juice was injected directly into the column, and anthocyanin pigments were eluted using 70% ethanol. The purified juice was subsequently concentrated by evaporating ethanol in a vacuum evaporator (bath temperature 40 °C, 239 mbar). The concentrated juices (anthocyanin pigments) were transferred onto Petri dishes and dried at room temperature for 24 h under a fume hood. The resulting powder was frozen at a temperature of −18 °C for further analysis [46].

#### 3.1.3. Lyophilization

Samples of potatoes (ca. 1 kg) and potato juices (ca. 1 L) were lyophilized in a Christ Alpha 1–4 LSCplus apparatus (Osterode am Hatz, Germany) at the following parameters: pressure 63 Pa, shelf heating temperature 30 °C, duration from 24 h (anthocyanin pigments) to 48 h (potato juices). The freeze-dried samples were stored at a temperature of −18 °C in closed containers in a dark place (in a freezer) until analyzed.

### 3.2. Model Study of Anthocyanin Pigment Extracts

#### 3.2.1. Determination of the Influence of Temperature on Anthocyanin Stability

Stock solutions of the pigments were prepared at the ratio of 0.5 g of pigment to 250 mL of water. Next, 10 mL portions of the resulting aqueous solutions of the pigments were poured into tubes, which were placed in a water bath at temperatures of 60 °C, 70 °C, 80 °C, 90 °C, and 100 °C for 5, 10, and 15 min. Samples were taken for analysis from each heating temperature and time variant.

#### 3.2.2. Determination of the Effect of pH on Anthocyanin Stability

The pH value of the aqueous solution of pigments (prepared as described above) was measured, and then the solution was poured into six 50 mL beakers. Next, 1 M NaOH or 7.5% HCl were added to the beakers to obtain solutions with pH = 1, pH = 3, pH = 5, pH = 7, pH = 9, and pH = 11. Once the solutions had reached the desired pH, their samples were collected for further analysis.

### 3.3. Preparation of Yoghurts

The yoghurts were prepared according to the method of Trigueros et al. [47] using the following yoghurt cultures: *Streptococcus thermophilus*, *Lactobacillus delbrueckii* subsp. *Lactis*, and *Lactobacillus delbrueckii* subsp. *bulgaricus* (CHOOZIT™ MY800 LYO 5 DCU. Rhodia Food-Danisco A/S. Sassenage, France). Whole UHT (Hacendado, Alicante, Spain) was heated in a water bath at a temperature of 43 °C for 30 min and inoculated with 50 g of industrial starter cultures per 1 L of milk. After inoculation, the milk was incubated at 43 °C, and the acidification curve was followed. Once the yoghurt had reached pH 4.7–4.8, it was cooled, and lyophilized pigment was added in the amount of 0.4 g/300 mL of yoghurt. Yoghurt without pigment addition served as the control sample. Samples to be analyzed were stored in a refrigerator at temperatures of 0 to 4 °C for 1 to 7 days.

### 3.4. Sensory Assessment

Quality descriptive analysis of the yogurts was conducted by nine trained panelists, each of whom had completed over 600 h of training in sensory analysis at the CIARGO Institute (UMH) (Group for Food Quality and Safety), and a staff member of the Wrocław University of Environmental and Life Sciences (Faculty of Biotechnology and Food Sciences. Department of Agricultural Technology and Storage). Prior to the assessment, the panelists cooperated in order to establish evaluation guidelines and a list of key sensory attributes based on the literature and own experience. The list included four major groups of attributes, visual, basic tastes, flavor, and texture, with individual specified attributes. The assessment was performed directly on fresh samples, served in transparent plastic cups with a lid. Sensory data were gathered from the panelists, who performed the assessment based on a descriptive scale (only for color description), as well as a mono-polar numerical scale ranging from 0 to 10 with 5 scales (where 0 denoted a lack of intensity and 10 meant exceptionally high intensity). The samples were coded with digits from 1 to 18 and compared with the control sample (C—yoghurt without additives).

### 3.5. Analytical Methods

#### 3.5.1. Potato Juices

The pasteurized juices with the addition of fruit and vegetable juice concentrates (characteristics of concentrates in Table S1) and the control samples (CSP CSR—non-pasteurized juices without the concentrates) were subjected to a color profile analysis with the calorimetric method (Table 1) [48]. In turn, the lyophilized samples were analyzed for the content of total polyphenols (TPs) (Table 2) [49,50], as well as the content and composition of anthocyanins (TAs), with liquid chromatography (HPLC-DAD and UHPLC MS/MS) (Table 2, Table 3 and Table 4) [51].

#### 3.5.2. Anthocyanin Pigments

The lyophilized pigments produced from potato juices were determined for the color profile with the calorimetric method (Table 5) [48], and the content of total polyphenols (TPs) (Table 2) [49,50] and the content and composition of anthocyanins (TAs) were determined with the HPLC-DAD and UHPLC MS/MS chromatography methods (Table 2, Table 6, and Table 7) [51]. Model studies (effect of temperature or pH) of aqueous solutions of anthocyanin pigment extracts: the color profile with the colorimetric method (Table 8, Table 10 and Table S2) [48], and the content of total polyphenols (TPs) (Table 9, Table 11, and Table S3) [49] and antioxidative activity with the ABTS+ (Tables S4 and S7) [52], DPPH (Tables S5 and S7) [52], and FRAP (Tables S6 and S7) [52] methods.

#### 3.5.3. Yoghurts

The yoghurts with the addition of lyophilized potato pigments and the control yoghurts (without the pigments) were analyzed for pH (Table 13) [53] and color with the calorimeter (Table 12 and Table S8) [48]. The analyses were conducted immediately after the preparation of yoghurts with pigments. Color analysis was performed every 15 sec for 6 min on day 1 (Table S8) [48], and then once a day on day 2 and 7 following yoghurt preparation (Table 11) [48]. The yoghurts were also subjected to microbiological analysis for lactic acid bacteria counts: Lactobacilli were estimated using MRS agar (37 °C, microaerophilia, 48 h) and lactococci M17 agar (30 °C, aerobiosis, 48 h) (Table 13) [53]. The microbiological analyses were performed 2 and 7 days after yoghurt preparation. The yoghurts with and without the addition of lyophilized anthocyanin pigments were also subjected to sensory analysis (Figure 1 and Figure 2) [47,54].

#### 3.5.4. Statistical Analysis

The results were processed via one-way and two-way analyses of variance using Statistica 13.1 package, and the least significant difference (LSD) and homogenous groups were determined with the Duncan test at a significance level of α = 0.05.

The content and composition of anthocyanins were determined in two laboratory replications, whereas polyphenol content determination and color analysis were conducted in six laboratory replications. The results presented in the manuscript represent the mean of the laboratory replications and 2 study years.

## 4. Conclusions

Both the pasteurization process and the addition of fruit and vegetable concentrates to potato juices positively affected their color and its stability in time. However, the pasteurized juices contained fewer biologically-active compounds than the unpasteurized and juices with concentrates. The anthocyanin pigments obtained from the juices of the red potato varieties were characterized by higher stability compared to the pigments from the purple juices. Based on modelling studies and when added to natural yoghurts, it has been shown that pigments obtained from potato juices are stable.

The organoleptic evaluation of the yogurts with additives showed that they had a more intense color and were less acidic compared to the natural yogurt. Undesirable off-flavors and spiciness were found in the yogurts enriched with pigment. However, when the pigments were mixed with fruit extracts, the sensory quality of the yogurts improved.

The results of the stability analysis of the acylated anthocyanins isolated from the potatoes indicate their great potential to be used in the future as improvers of not only the quality of food products (their color, in particular), but also their nutritional value.

## Figures and Tables

**Table 1:** Values of the L*, a*, and b* parameters from potato juices of red- and purple-fleshed varieties: non-pasteurized and without additives (control sample CS); after pasteurization at temperatures of 65 °C and 75 °C; with addition of lemon (Le), lime (Li), and rhubarb (Rh) concentrates.

Flesh Color	Variety	Variant	0 h			1 h			4 h		
			L*	a*	b*	L*	a*	b*	L*	a*	b*
purple	Provita	CS	0.61 ^aB^	3.32 ^aC^	1.25 ^aC^	0.34 ^aA^	1.73 ^aAB^	0.44 ^aB^	0.30 ^aA^	1.61 ^aA^	0.33 ^aA^
		65 °C	4.36 ^bA^	5.38 ^cA^	11.6 ^bA^	20.1 ^cC^	34.4 ^dC^	32.1 ^bC^	16.9 ^cB^	31.7 ^cB^	27.8 ^bB^
		75 °C	5.63 ^bA^	4.31 ^cA^	12.0 ^bA^	21.4 ^cC^	35.5 ^dC^	33.3 ^bC^	19.5 ^dB^	33.6 ^cB^	3.8 ^cB^
		Le	2.34 ^aB^	2.93 ^aA^	1.54 ^aA^	1.78 ^abA^	6.51 ^bC^	1.95 ^aB^	1.75 ^bA^	6.20 ^bB^	2.33 ^aC^
		Li	2.34 ^aB^	9.80 ^bB^	2.38 ^aA^	3.19 ^bC^	11.8 ^cC^	4.12 ^aC^	1.80 ^bA^	7.05 ^bA^	2.49 ^aB^
		Rh	0.12 ^aA^	0.93 ^aA^	0.57 ^aA^	1.22 ^abB^	5.44 ^abB^	1.37 ^aB^	1.70 ^bC^	6.37 ^bC^	2.30 ^aC^
	Double Fun	CS	0.19 ^aB^	0.23 ^aA^	−0.05 ^aA^	0.16 ^aA^	0.28 ^aAB^	0.05 ^aB^	0.15 ^aA^	0,36 ^aB^	0.03 ^aB^
		65 °C	1.94 ^dB^	8.14 ^dB^	1.38 ^cA^	1.88 ^dA^	7.32 ^dA^	1.53 ^dB^	1.89 ^cA^	8.46 ^deC^	1.92 ^dC^
		75 °C	2.25 ^eB^	9.80 ^eC^	1.62 ^dA^	1.94 ^dA^	7.90 ^dA^	1.67 ^dA^	2.09 ^cAB^	9.42 ^eB^	2.17 ^dB^
		Le	0.72 ^cA^	3.01 ^cA^	0.39 ^bA^	0.85 ^cAB^	4.28 ^cB^	0.77 ^cAB^	1.22 ^bB^	6.29 ^cdC^	1.34 ^cB^
		Li	0.45 ^bA^	1.80 ^bA^	0.26 ^bA^	0.60 ^bcAB^	2.90 ^bB^	0.48 ^bAB^	0.90 ^bB^	4.55 ^bcC^	0.87 ^bcB^
		Rh	0.46 ^bA^	1.28 ^bA^	−0.01 ^aA^	0.54 ^bAB^	1.80 ^bB^	0.33 ^bB^	0.81 ^bB^	3.48 ^bC^	0.74 ^bC^
red	Lily Rose	CS	0.56 ^aB^	3.06 ^aC^	0.92 ^aC^	0.44 ^aAB^	2.58 ^aB^	0.73 ^aB^	0.32 ^aA^	1.70 ^aA^	0.45 ^aA^
		65 °C	28.3 ^cB^	36.3 ^eC^	36.1 ^dB^	25.5 ^dAB^	34.8 ^deB^	38.7 ^dC^	18.5 ^dA^	29.9 ^cdA^	30.0 ^dA^
		75 °C	32.1 ^cC^	38.3 ^eC^	36.5 ^dAB^	29.2 ^dB^	36.6 ^eB^	40.7 ^eB^	22.0 ^eA^	32.2 ^cdA^	35.0 ^dA^
		Le	7.67 ^bB^	23.4 ^bB^	9.81 ^bC^	5.19 ^bA^	21.0 ^bA^	6.11 ^bA^	5.75 ^bAB^	23.2 ^bB^	7.04 ^abB^
		Li	8.12 ^bB^	27.2 ^cBC^	10.8 ^bB^	6.56 ^bA^	24.5 ^cA^	8.44 ^bA^	10.3 ^bcC^	28.6 ^cC^	14.3 ^bcC^
		Rh	12.2 ^bA^	32.0 ^dA^	17.5 ^cA^	13.2 ^cB^	32.8 ^dB^	19.2 ^cB^	12.7 ^cAB^	32.8 ^dB^	18.5 ^cAB^

Data are expressed as the mean, *n* = 12. Results in the same column followed by different letters indicate significant differences according to Duncan’s test at *p* < 0.05 between different flesh colors and varieties (small letters), and between times (big letters), as determined by one-way ANOVA.

**Table 2:** Contents of total polyphenols (TPs) (mg GAE/g DM) and anthocyanins (TAs) (mg/100 g DM) in potato juices and pigment: non-pasteurized and without additives (control samples (CSs)); after pasteurization at temperatures of 65 °C and 75 °C; with addition of lemon (Le), lime (Li), and rhubarb (Rh) concentrates.

Flesh Color	Variety	Variant	Juice		Pigment	
			TP	TA	TP	TA
purple	Provita	CS	7.26 ± 0.15	27.8 ± 0.13	138.2 ± 0.10	1098.1 ± 0.10
		65 °C	3.95 ± 0.13	15.8 ± 0.15	160.4 ± 0.10	1321.5 ± 0.13
		75 °C	4.54 ± 0.12	12.2 ± 0.12	164.3 ± 0.10	1343.9 ± 0.13
		Le	10.7 ± 0.14	54.7 ± 0.14	152.6 ± 0.01	4279.9 ± 0.10
		Li	14.6 ± 0.16	109 ± 0.11	121.5 ± 0.01	3707.8 ± 0.30
		Rh	11.7 ± 0.12	118 ± 0.12	156.3 ± 0.01	4499.1 ± 0.14
	Double Fun	CS	9.45 ± 0.13	70.6 ± 0.07	147.2 ± 0.01	5487.8 ± 0.15
		65 °C	13.2 ± 0.16	37.4 ± 0.17	163.8 ± 0.01	1084.8 ± 0.10
		75 °C	9.83 ± 0.10	7.50 ± 0.05	166.6 ± 0.01	1095.6 ± 0.09
		Le	17.3 ± 0.17	117.8 ± 0.17	155.4 ± 0.10	5878.5 ± 0.15
		Li	16.3 ± 0.15	146.6 ± 0.14	125.5 ± 0.01	5460.6 ± 0.50
		Rh	13.8 ± 0.19	130.9 ± 0.13	158.8 ± 0.01	6368.3 ± 0.50
red	Lily Rose	CS	7.32 ± 0.18	13.9 ± 0.12	115.5 ± 0.01	1157.2 ± 0.12
		65 °C	2.69 ± 0.19	14.4 ± 0.14	97.7 ± 0.51	1474.6 ± 0.14
		75 °C	3.65 ± 0.16	9.40 ± 0.09	114.9 ± 0.01	1722.7 ± 0.17
		Le	20.5 ± 0.11	152.0 ± 0.15	59.4 ± 0.01	5934.3 ± 0.52
		Li	23.0 ± 0.18	145.6 ± 0.14	92.2 ± 0.01	5562.6 ± 0.53
		Rh	15.2 ± 0.17	89.8 ± 0.09	129.4 ± 0.01	6225.1 ± 0.51
		LSD	5.18	9.00	21.0	100

Data are expressed as the mean, *n* = 12, and standard deviation (±SD). LSD—last significant difference.

**Table 3:** Content of identified anthocyanins (mg/100 g DM) in purple-fleshed potato juices: non-pasteurized and without additives (control samples (CSs)); after pasteurization at temperatures of 65 °C and 75 °C; with addition of lemon (Le), lime (Li), and rhubarb (Rh) concentrates.

Compound	Provita						Double Fun					
	CS	65 °C	75 °C	Le	Li	Rh	CS	65 °C	75 °C	Le	Li	Rh
Petunidin 3-rutinoside 5-glucoside	0.72	0.51	0.53	1.94	3.96	3.54	0.64	-	-	-	-	-
Peonidin 3-rutinoside 5-glucoside isomer 1	0.39	0.28	0.27	-	-	-	-	-	-	2.96	3.32	2.69
Peonidin 3-rutinoside 5-glucoside isomer 2	-	-	-	-	-	-	-	-	-	2.09	2.34	2.21
Petunidin 3-caffeoylrutinoside 5-glucoside isomer 1	-	-	-	-	-	-	-	-	-	0.72	0.83	0.81
Petunidin 3-caffeoylrutinoside 5-glucoside isomer 2	-	-	-	-	-	-	-	-	-	1.03	1.30	1.33
Delphinidin 3-coumaroylrutinoside 5-glucoside	0.83	0.40	0.44	0.95	1.09	1.48	-	-	-	-	-	-
Malvidin derivative	-	-	-	-	-	-	0.84	0.58	-	0.81	0.84	0.87
Petunidin 3-coumaroylrutinoside 5-glucoside	8.61	3.39	2.38	12.8	21.9	33.7	12.8	4.61	-	3.97	4.86	7.16
Malvidin 3-coumarylorutinoside 5-glucoside isomer 1	-	-	-	-	-	-	60.9	33.0	7.50	102.7	129.2	112.8
Malvidin 3-feruloylrutinoside 5-glucoside	-	-	-	-	-	-	4.56	2.81	-	3.37	3.77	3.72
Malvidin 3-coumarylorutinoside 5-glucoside isomer 2	-	-	-	-	-	-	0.55	0.77	-	0.27	0.24	0.25
Petunidin 3-feruloylrutinoside 5-glucoside	0.64	0.45	0.49	0.61	1.13	2.01	-	-	-	-	-	-
Peonidin 3-coumaroylrutinoside 5-glucoside	15.7	10.0	7.25	36.8	77.1	73.5	-	-	-	-	-	-
Peonidin 3-feruloylrutinoside 5-glucoside	0.89	0.70	0.50	1.53	3.82	3.84	-	-	-	-	-	-
Peonidin derivative	-	-	0.30	-	-	-	-	-	-	-	-	-

**Table 4:** Content of identified anthocyanins (mg/100 g DM) in red-fleshed potato juices: non-pasteurized and without additives (control samples (CSs)); after pasteurization at temperatures of 65 °C and 75 °C; with addition of lemon (Le), lime (Li), and rhubarb (Rh) concentrates.

Compound	Lily Rose					
	CS	65 °C	75 °C	Le	Li	Rh
Pelargonidin 3-rutinoside 5-glucoside	0.80	0.64	0.47	12.6	12.8	7.61
Pelargonidin derivative 1	0.59	0.41	0.33	1.44	1.24	0.96
Pelargonidin derivative 2	0.40	0.29	-	1.08	1.06	0.75
Pelargonidin 3-coumaroylrutinoside 5-glucoside isomer 1	0.41	0.68	0.37	-	-	-
Pelargonidin 3-caffeoylrutinoside 5-glucoside isomer 1	0.64	0.75	0.53	4.19	3.68	2.69
Pelargonidin 3-caffeoylrutinoside 5-glucoside isomer 2	0.38	-	-	-	-	-
Unidentified	0.29	-	-	4.63	4.65	3.07
Pelargonidin 3-coumaroylrutinoside 5-glucoside isomer 2	1.19	0.72	0.48	3.87	3.49	2.35
Pelargonidin 3-coumaroylrutinoside 5-glucoside isomer 3	7.73	9.40	5.71	116.8	112.0	67.9
Pelargonidin 3-feruloylrutinoside 5-glucoside	0.64	0.75	0.50	7.36	6.80	4.42
Pelargonidin 3-coumaroylrutinoside 5-glucoside isomer 4	0.93	0.36	0.41	-	-	-
Pelargonidin 3-coumaroylrutinoside 5-glucoside isomer 5	-	0.32	0.49	-	-	-
Pelargonidin 3-coumaroylrutinoside 5-glucoside isomer 6	-	0.75	0.74	-	-	-

**Table 5:** Values of the L*, a*, and b* parameters from lyophilized pigments of potato juices of red- and purple-fleshed varieties: non-pasteurized and without additives (control sample CS); after pasteurization in temperature 65 °C and 75 °C; with additives of lemon (Le), lime (Li), and rhubarb (Rh) concentrates.

Flesh Color	Variety	Variant	0 h			1 h			4 h		
			L*	a*	b*	L*	a*	b*	L*	a*	b*
purple	Provita	Cs	36.3 ^eA^	2.70 ^eA^	−3.57 ^aA^	36.2 ^eA^	2.45 ^dA^	−3.51 ^aA^	36.6 ^fA^	2.50 ^dA^	−3.60 ^aA^
		65 °C	32.7 ^aA^	0.12 ^bA^	−1.00 ^cA^	32.8 ^aA^	0.06 ^aA^	−0.88 ^dA^	32.6 ^aA^	0.13 ^aA^	−0.95 ^cA^
		75 °C	33.3 ^bA^	0.05 ^aA^	−1.02 ^cA^	33.3 ^bA^	0.08 ^aA^	−1.00 ^cA^	33.3 ^cA^	0.14 ^aB^	−1.00 ^cA^
		Le	34.5 ^dA^	0.40 ^cA^	−0.86 ^dA^	34.6 ^dA^	0.35 ^bA^	−0.85 ^dA^	34.5 ^eA^	0.37 ^bA^	−0.95 ^cA^
		Li	34.1 ^cA^	0.45 ^cA^	−0.76 ^eA^	34.3 ^cA^	0.39 ^bA^	−0.71 ^eA^	34.3 ^dA^	0.42 ^bA^	−0.69 ^dA^
		Rh	32.8 ^aA^	0.96 ^dA^	−1.31 ^bA^	32.8 ^aA^	0.98 ^cA^	−1.30 ^bA^	32.7 ^bA^	0.99 ^cA^	−1.32 ^bA^
	Double Fun	CS	33.0 ^aA^	0.10 ^bA^	−1.28 ^bA^	33.1 ^aA^	0.07 ^bA^	−1.23 ^bA^	33.2 ^bA^	0.17 ^bA^	−1.32 ^bA^
		65 °C	34.1 ^eA^	−0.01 ^aA^	−1.26 ^bA^	34.1 ^cA^	−0.07 ^aA^	−1.15 ^cA^	34.0 ^dA^	−0.05 ^aA^	−1.21 ^cA^
		75 °C	33.8 ^dA^	0.47 ^cA^	−1.43 ^aA^	33.8 ^bA^	0.47 ^cA^	−1.40 ^aA^	33.5 ^cA^	0.47 ^cA^	−1.47 ^aA^
		Le	34.3 ^fA^	0.86 ^dA^	−0.07 ^eA^	34.1 ^cA^	0.83 ^dA^	−0.18 ^eA^	34.5 ^eA^	0.87 ^dA^	−0.15 ^eA^
		Li	33.5 ^cA^	1.03 ^eA^	−0.92 ^dA^	33.1 ^aA^	0.85 ^dA^	−0.97 ^dA^	33.0 ^bA^	0.98 ^eA^	−0.97 ^dA^
		Rh	33.3 ^bA^	1.08 ^eA^	−1.17 ^cA^	33.0 ^aA^	1.06 ^eA^	−1.14 ^cA^	32.8 ^aA^	0.93 ^deA^	−1.14 ^cA^
red	Lily Rose	CS	36.6 ^dA^	4.18 ^dA^	−0.31 ^bA^	36.6 ^dA^	4.34 ^dA^	−0.11 ^bA^	36.8 ^dA^	4.54 ^cA^	−0.12 ^cA^
		65 °C	35.3 ^cB^	1.84 ^bA^	−0.74 ^aA^	35.3 ^cB^	1.81 ^aA^	−0.68 ^aA^	34.9 ^bA^	2.02 ^aA^	−0.54 ^aA^
		75 °C	35.1 ^bB^	1.54 ^aA^	−0.82 ^aA^	34.9 ^bA^	1.92 ^bA^	−0.64 ^aA^	34.9 ^bA^	2.04 ^aA^	−0.57 ^aA^
		Le	33.7 ^aA^	2.78 ^cA^	−0.38 ^bA^	33.9 ^aA^	2.83 ^cA^	−0.17 ^bA^	33.8 ^aA^	2.86 ^bA^	−0.27 ^bA^
		Li	36.6 ^dA^	12.1 ^eA^	1.62 ^cA^	36.7 ^dA^	12.3 ^eA^	1.75 ^cA^	36.7 ^cA^	12.5 ^dA^	1.72 ^dA^
		Rh	41.1 ^eA^	22.7 ^fA^	3.50 ^dA^	41.3 ^eA^	23.1 ^fA^	3.58 ^dA^	41.3 ^eA^	23.0 ^eA^	3.57 ^eA^

Data are expressed as the mean, *n* = 12. Results in the same column followed by different letters indicate significant differences according to Duncan’s test at *p* < 0.05 between different variants in varieties (small letters); results in the same line followed by different letters indicate significant differences according to Duncan’s test at *p* < 0.05 between times (big letters), as determined by one-way ANOVA.

**Table 6:** Content of identified anthocyanins (mg/100 g DM) in lyophilized pigments of purple-fleshed potato juices: non-pasteurized and without additives (control samples (CSs)); after pasteurization at temperatures of 65 °C and 75 °C; with addition of lemon (Le), lime (Li), and rhubarb (Rh) concentrates.

Compound	Provita						Double Fun					
	CS	65 °C	75 °C	Le	Li	Rh	CS	65 °C	75 °C	Le	Li	Rh
Petunidin3-rutinoside 5-glucoside	80.6	87.2	85.5	393.9	362.0	373.2	191.1	-	-	-	-	-
Peonidin3-rutinoside 5-glucoside isomer 1	33.0	57.1	61.3	263.9	265.6	264.8	190.1	-	-	342.5	336.6	337.7
Peonidin3-rutinoside 5-glucoside isomer 2	-	-	-	-	-	-	-	-	-	320.7	312.6	327.3
Petunidin 3-caffeoylrutinoside 5-glucoside isomer 2	-	-	-	-	-	-	101.0	-	-	281.0	278.7	282.7
Petunidin 3-caffeoylrutinoside 5-glucoside isomer 1	-	-	-	-	-	-	141.2	96.1	103.8	333.3	327.3	348.4
Delphinidin 3-coumaroylrutinoside 5-glucoside	81.1	31.1	35.3	266.3	262.4	275.0	-	-	-	-	-	-
Malvidin derivative	-	-	-	-	-	-	115.6	92.5	93.2	299.3	298.7	309.3
Petunidin 3-coumaroylrutinoside 5-glucoside	338.1	243.8	260.6	703.9	544.5	839.2	344.7	110.9	118.1	379.4	347.7	402.3
Malvidin 3-coumarylorutinoside 5-glucoside isomer 1	-	-	-	-	-	-	4118.4	785.3	780.5	3596.7	3244.9	4014.9
Malvidin 3-feruloylrutinoside 5-glucoside	-	-	-	-	-	-	285.7	-	-	325.6	314.1	345.7
Petunidin 3-feruloylrutinoside 5-glucoside	58.2	58.4	59.7	266.3	262.9	271.9	-	-	-	-	-	-
Peonidin 3-coumaroylrutinoside 5-glucoside	503.7	835.8	834.9	2065.5	1707.4	2149.3	-	-	-	-	-	-
Peonidin 3-feruloylrutinoside 5-glucoside	39.8	47.2	48.6	320.0	303.0	325.6	-	-	-	-	-	-

**Table 7:** Content of identified anthocyanins (mg/100 g DM) in lyophilized pigments of red-fleshed potato juices: non-pasteurized and without additives (control samples (CSs)); after pasteurization at temperatures of 65 °C and 75 °C; with addition of lemon (Le), lime (Li), and rhubarb (Rh) concentrates.

Compound	Lily Rose					
	CS	65 °C	75 °C	Le	Li	Rh
Pelargonidin3-rutinoside 5-glucoside	121.6	137.8	159.57	632.35	611.57	646.27
Unidentified	-	-	-	97.20	106.17	-
Pelargonidin derivative 1	-	95.4	101.27	286.52	281.07	284.98
Pelargonidin derivative 2	-	99.4	107.61	277.33	270.20	273.43
Pelargonidin 3-coumaroylrutinoside 5-glucoside isomer 1	-	114.8	140.78	-	-	-
Pelargonidin3-caffeoylrutinoside 5-glucoside isomer 1	98.3	118.9	132.56	334.32	312.48	345.73
Pelargonidin3-caffeoylrutinoside 5-glucoside isomer 2	100.5	104.7	111.71	-	-	-
unidentified	102.4	-	-	366.33	368.06	366.48
Pelargonidin 3-coumaroylrutinoside 5-glucoside isomer 2	108.1	-	-	335.15	318.81	346.21
Pelargonidin 3-coumaroylrutinoside 5-glucoside isomer 3	517.4	684.9	842.32	3034.61	2744.79	3321.29
Pelargonidin3-feruloylrutinoside 5-glucoside	108.9	118.7	126.86	398.7605	381.93	418.17
Pelargonidin 3-coumaroylrutinoside 5-glucoside isomer 4	-	-	-	440.772	441.24	445.22

**Table 8:** Model study of color change L*, a*, and b* parameters under the influence of temperature from 60 °C to 100 °C after 5, 10, and 15 min (the table presents averages from time) in pigment extracts of purple or red potato juices: non-pasteurized and without additives (control sample (CSs)); after pasteurization at temperatures of 65 °C and 75 °C; with addition of lemon (Le), lime (Li), and rhubarb (Rh) concentrates.

Temperature	Color Parameters	Purple						Red					
		CS	65 °C	75 °C	Le	Li	Rh	CS	65 °C	75 °C	Le	Li	Rh
Non-pasteurized	L*	5.49 ^bA^	21.0 ^eA^	1.79 ^aA^	10.8 ^cA^	14.3 ^dA^	5.30 ^bA^	70.2 ^hC^	68.4 ^hD^	65.6 ^gE^	69.6 ^iE^	77.7 ^jD^	63.1 ^fA^
	a*	17.9 ^gA^	23.8 ^hB^	6.43 ^aA^	31.0 ^kF^	30.1 ^jF^	25.9 ^iE^	8.11 ^bB^	10.7 ^cA^	8.79 ^bA^	14.6 ^eB^	12.9 ^dB^	16.3 ^fA^
	b*	4.18 ^eA^	26.8 ^hA^	0.42 ^dA^	−20.3 ^bA^	−18.5 ^cA^	−25.7 ^aA^	29.4 ^jA^	39.1 ^lA^	37.0 ^kA^	15.5 ^gA^	13.9 ^fA^	28.3 ^iA^
60 °C	L*	24.9 ^bB^	34.2 ^dB^	12.1 ^aB^	38.6 ^eB^	38.2 ^eB^	25.2 ^cB^	64.5 ^gB^	64.9 ^gC^	61.7 ^fD^	66.3 ^hC^	73.8 ^iA^	64.8 ^gB^
	a*	19.9 ^jB^	20.7 ^kA^	19.7 ^jC^	6.8 ^cE^	5.53 ^bE^	−3.71 ^aD^	8.47 ^dB^	11.4 ^fB^	9.34 ^eB^	14.6 ^hB^	12.0 ^gB^	18.3 ^iB^
	b*	35.1 ^hB^	49.7 ^kB^	16.1 ^dB^	8.96 ^bB^	10.0 ^cB^	−6.46 ^aB^	32.3 ^gB^	40.9 ^jB^	39.3 ^iB^	21.1 ^fC^	19.8 ^eB^	32.3 ^gB^
70 °C	L*	32.9 ^cC^	36.1 ^dC^	25.9 ^aC^	40.4 ^eC^	40.1 ^fC^	31.3 ^bC^	63.9 ^gA^	64.4 ^gC^	59.6 ^hC^	71.9 ^jF^	75.7 ^kC^	68.7 ^iD^
	a*	19.6 ^hB^	20.9 ^iA^	18.9 ^gB^	3.11 ^cD^	1.94 ^bD^	−9.56 ^aC^	7.96 ^dA^	11.8 ^fB^	9.36 ^eB^	11.6 ^fA^	11.6 ^fA^	18.1 ^gB^
	b*	48.1 ^jC^	54.6 ^kC^	39.0 ^hC^	11.8 ^bC^	12.2 ^cC^	2.03 ^aC^	34.0 ^gC^	41.7 ^iC^	40.9 ^iC^	16.3 ^dB^	19.9 ^eB^	32.5 ^fB^
80 °C	L*	34.4 ^cD^	36.9 ^dC^	29.3 ^aD^	40.4 ^eC^	41.2 ^fD^	31.9 ^bC^	63.2 ^iA^	63.1 ^iB^	58.7 ^gB^	61.8 ^hA^	74.6 ^kB^	68.3 ^jD^
	a*	19.8 ^iB^	20.8 ^jA^	19.3 ^iC^	1.90 ^cC^	0.92 ^bC^	−12.3 ^aA^	8.40 ^dB^	12.3 ^fC^	9.81 ^eB^	17.1 ^hD^	13.5 ^gC^	19.0 ^iC^
	b*	51.3 ^iD^	56.0 ^jD^	44.2 ^hD^	12.9 ^bD^	14.4 ^cD^	5.65 ^aD^	34.9 ^fC^	42.7 ^gD^	42.1 ^gD^	26.2 ^eD^	21.1 ^dC^	34.8 ^fC^
90 °C	L*	38.5 ^cE^	38.8 ^cD^	33.5 ^aE^	41.8 ^dD^	43.7 ^eE^	37.1 ^bD^	63.3 ^hA^	62.6 ^gA^	57.5 ^fA^	62.5 ^gB^	74.3 ^jB^	67.9 ^iC^
	a*	21.2 ^jC^	23.5 ^kB^	20.4 ^iD^	1.62 ^cB^	−1.5 ^bB^	−10.9 ^aB^	8.97 ^dB^	12.5 ^fC^	10.8 ^eC^	16.6 ^hC^	13.5 ^gC^	19.8 ^iD^
	b*	57.4 ^hE^	58.9 ^iE^	50.9 ^gE^	16.6 ^aE^	16.5 ^aE^	15.9 ^aE^	37.2 ^eD^	43.1 ^fE^	43.3 ^fE^	27.2 ^cE^	21.8 ^bC^	35.8 ^dD^
100 °C	L*	38.3 ^cE^	38.2 ^cD^	34.0 ^aF^	42.7 ^dE^	43.4 ^eE^	37.3 ^bD^	63.5 ^gA^	63.2 ^gB^	58.6 ^fB^	67.3 ^hD^	74.7 ^jB^	68.3 ^iD^
	a*	21.1 ^jC^	23.6 ^kB^	19.8 ^iC^	−2.12 ^bA^	−2.09 ^cA^	−12.9 ^aA^	9.47 ^dC^	12.5 ^fC^	11.6 ^eD^	14.9 ^hB^	13.7 ^gC^	20.3 ^iE^
	b*	57.4 ^iE^	58.3 ^jE^	50.9 ^hE^	17.8 ^bF^	19.2 ^cF^	15.7 ^aE^	38.3 ^fE^	43.2 ^gE^	42.9 ^gD^	26.4 ^eD^	23.7 ^dD^	37.1 ^fE^

Data are expressed as the mean, *n* = 6. Results in the same line followed by different letters indicate significant differences according to Duncan’s test at *p* < 0.05 between different variants in flesh color (small letters); results in the same column followed by different letters indicate significant differences according to Duncan’s test at *p* < 0.05 between different temperatures (big letters), as determined by one-way ANOVA.

**Table 9:** Model study content of total polyphenols (TPs) (mg GAE/g DM), as affected by temperature changes from 60 °C to 100 °C in pigment extracts of purple or red potato juices: non-pasteurized and without additives (control samples (CSs)); after pasteurization at temperatures of 65 °C and 75 °C; with addition of lemon (Le), lime (Li), and rhubarb (Rh) concentrates.

Color Flesh	Variant	Non-Pasteurized	60 °C	70 °C	80 °C	90 °C	100 °C
purple	CS	150.1 ± 0.01	145.7 ± 0.01	161.0 ± 0.01	157.3 ± 0.15	147.8 ± 0.15	157.3 ± 0.15
	65 °C	158.0 ± 0.01	154.9 ± 0.01	144.9 ± 0.15	139.8 ± 0.15	146.6 ± 0.15	151.2 ± 0.15
	75 °C	167.5 ± 0.01	166.1 ± 0.01	161.7 ± 0.01	161.5 ± 0.15	158.3 ± 0.15	157.2 ± 0.15
	Le	154.4 ± 0.01	143.8 ± 0.05	143.1 ± 0.05	143.5 ± 0.05	142.9 ± 0.01	136.4 ± 0.01
	Li	130.3 ± 0.01	121.1 ± 0.05	111.1 ± 0.05	132.7 ± 0.05	135.4 ± 0.01	136.1 ± 0.01
	Rh	152.1 ± 0.01	141.0 ± 0.01	111.4 ± 0.05	146.8 ± 0.05	141.9 ± 0.05	147.7 ± 0.01
red	CS	108.4 ± 0.01	100.2 ± 0.01	101.5 ± 0.01	78.2 ± 0.01	100.8 ± 0.01	102.3 ± 0.01
	65 °C	97.1 ± 0.01	108.3 ± 0.01	99.5 ± 0.01	97.5 ± 0.01	97.6 ± 0.01	99.1 ± 0.01
	75 °C	115.7 ± 0.01	109.4 ± 0.01	109.8 ± 0.01	116.7 ± 0.01	109.5 ± 0.01	109.6 ± 0.1
	Le	56.9 ± 0.15	140.8 ± 0.15	141.2 ± 0.05	93.2 ± 0.01	145.0 ± 0.01	105.3 ± 0.01
	Li	110.8 ± 0.15	118.3 ± 0.15	132.5 ± 0.15	114.2 ± 0.01	130.4 ± 0.01	112.8 ± 0.01
	Rh	145.9 ± 0.01	145.1 ± 0.05	121.9 ± 0.05	132.8 ± 0.01	106.9 ± 0.01	146.4 ± 0.01

Data are expressed as the mean, *n* = 6, and standard deviation (±SD).

**Table 10:** Model study of color change L*, a*, and b* parameters under the influence of pH from 1 to 11 in pigment extracts of purple or red potato juices: non-pasteurized and without additives (control samples (CSs)); after pasteurization at temperatures of 65 °C and 75 °C; with addition of lemon (Le), lime (Li), and rhubarb (Rh) concentrates.

pH	Color Parameters	Purple						Red					
		CS	65 °C	75 °C	Le	Li	Rh	CS	65 °C	75 °C	Le	Li	Rh
1	L*	28.6 ^bC^	33.2 ^dD^	25.5 ^aE^	33.9 ^dB^	34.1 ^eD^	32.4 ^cC^	62.9 ^hC^	62.0 ^hC^	62.0 ^hC^	52.5 ^fC^	57.5 ^gB^	57.7 ^gD^
	a*	60.5 ^dF^	63.0 ^fF^	57.9 ^cF^	66.2 ^hF^	66.3 ^hF^	65.9 ^gF^	56.3 ^bE^	51.8 ^aF^	51.9 ^aF^	61.6 ^eE^	60.7 ^dE^	63.7 ^fF^
	b*	40.2 ^dE^	38.2 ^cE^	39.9 ^cdF^	25.8 ^aD^	25.6 ^aD^	29.1 ^bD^	55.4 ^gD^	53.8 ^fD^	50.4 ^eD^	80.8 ^iF^	79.4 ^hE^	82.1 ^jF^
3	L*	29.8 ^bD^	38.9 ^fE^	22.9 ^aD^	34.6 ^dC^	32.9 ^cC^	36.4 ^eD^	67.8 ^jD^	65.9 ^iD^	68.6 ^kE^	52.0 ^gC^	58.7 ^hC^	57.9 ^hD^
	a*	52.4 ^fE^	50.9 ^dE^	51.2 ^eE^	55.8 ^gE^	60.5 ^iE^	60.2 ^iE^	41.9 ^cD^	23.9 ^aE^	27.6 ^bE^	57.9 ^hD^	52.9 ^fD^	60.2 ^iE^
	b*	12.6 ^dD^	22.0 ^fC^	20.6 ^eD^	−0.46 ^bB^	6.80 ^cB^	−9.24 ^aB^	31.5 ^gB^	33.3 ^iA^	32.5 ^hA^	64.9 ^kD^	51.9 ^jC^	64.3 ^kD^
5	L*	32.7 ^dF^	45.6 ^fF^	25.1 ^cE^	36.3 ^eD^	22.1 ^bA^	11.7 ^aA^	70.9 ^kF^	71.7 ^kF^	68.9 ^iE^	66.1 ^gE^	67.6 ^hE^	69.6 ^jE^
	a*	47.6 ^iD^	32.9 ^gD^	50.4 ^jD^	53.4 ^kD^	−25.2 ^aB^	−14.8 ^bC^	8.44 ^cC^	12.9 ^eD^	22.8 ^fD^	12.9 ^eC^	9.39 ^dB^	39.7 ^hD^
	b*	9.89 ^dC^	25.8 ^hD^	10.9 ^eC^	−3.23 ^bA^	−1.61 ^cA^	−16.0 ^aA^	29.0 ^iA^	33.2 ^kA^	32.1 ^jA^	18.9 ^gA^	19.5 ^gA^	15.9 ^fA^
7	L*	16.9 ^cB^	18.6 ^dB^	16.5 ^aB^	20.7 ^eA^	23.1 ^fB^	12.4 ^bB^	70.3 ^kE^	69.3 ^jE^	67.7 ^iD^	61.9 ^hD^	74.4 ^lF^	56.0 ^gC^
	a*	35.3 ^jC^	21.7 ^iC^	6.49 ^dB^	−40.5 ^aA^	−33.8 ^cA^	−34.9 ^bA^	8.04 ^eC^	10.5 ^gC^	10.0 ^gC^	10.3 ^gA^	9.92 ^fB^	11.9 ^hA^
	b*	3.94 ^bB^	20.1 ^eB^	−0.56 ^aA^	6.30 ^dC^	24.7 ^fC^	4.48 ^cC^	29.0 ^gA^	38.9 ^iB^	35.8 ^hB^	21.2 ^eB^	20.9 ^eB^	40.4 ^jB^
9	L*	2.36 ^bA^	7.34 ^cA^	1.29 ^aA^	39.4 ^fE^	38.0 ^eE^	36.3 ^dD^	44.1 ^hA^	46.9 ^iA^	42.6 ^gA^	46.1 ^iA^	53.9 ^jA^	44.2 ^hA^
	a*	0.49 ^fA^	2.69 ^hA^	0.87 ^gA^	−15.9 ^cB^	−18.5 ^bC^	−32.9 ^aB^	−2.34 ^dA^	5.81 ^iA^	−1.30 ^eA^	11.6 ^kB^	10.8 ^jC^	20.3 ^lC^
	b*	2.30 ^bA^	9.89 ^cA^	0.74 ^aB^	52.2 ^jE^	50.2 ^iE^	42.7 ^fE^	32.1 ^dC^	43.9 ^gC^	37.6 ^eC^	45.9 ^hC^	58.2 ^kD^	58.9 ^lC^
11	L*	30.3 ^bE^	32.6 ^cC^	17.6 ^aC^	60.4 ^iF^	59.8 ^hF^	57.3 ^fE^	60.9 ^jB^	58.8 ^gB^	57.3 ^fB^	48.3 ^dB^	65.7 ^kD^	54.1 ^eB^
	a*	14.6 ^hB^	19.2 ^jB^	7.94 ^dC^	5.26 ^cC^	3.96 ^bD^	−7.36 ^aD^	4.61 ^bB^	9.53 ^fB^	7.82 ^dB^	12.2 ^gC^	8.69 ^eA^	17.4 ^iB^
	b*	50.2 ^bF^	54.6 ^cF^	28.9 ^aE^	85.3 ^jF^	83.6 ^iF^	77.2 ^gF^	66.9 ^dE^	69.6 ^fE^	67.7 ^eE^	67.2 ^deE^	89.4 ^kF^	80.9 ^hE^

Data are expressed as the mean, *n* = 6. Results in the same line followed by different letters indicate significant differences according to Duncan’s test at *p* < 0.05 between different variants in flesh color (small letters); results in the same column followed by different letters indicate significant differences according to Duncan’s test at *p* < 0.05 between different pH (big letters), as determined by one-way ANOVA.

**Table 11:** Model study content of total polyphenols (TPs) (mg GAE/g DM), as affected by pH from 1 to 11 in pigment extracts of purple or red potato juices: non-pasteurized and without additives (control samples (CSs); after pasteurization at temperatures of 65 °C and 75 °C; with addition of lemon (Le), lime (Li), and rhubarb (Rh) concentrates.

Color Flesh	Variant	1	3	5	7	9	11
purple	CS	214.2 ± 0.01	205.4 ± 0.01	216.7 ± 0.01	203.6 ± 0.01	192.3 ± 0.01	169.2 ± 0.01
	65 °C	201.7 ± 0.01	201.1 ± 0.01	191.1 ± 0.01	197.3 ± 0.01	164.8 ± 0.01	171.7 ± 0.01
	75 °C	203.6 ± 0.01	213.6 ± 0.01	208.6 ± 0.01	209.8 ± 0.01	183.6 ± 0.01	164.8 ± 0.01
	Le	163.6 ± 0.01	177.3 ± 0.01	180.4 ± 0.01	146.7 ± 0.01	136.1 ± 0.01	111.1 ± 0.01
	Li	166.1 ± 0.01	166.7 ± 0.01	163.6 ± 0.01	146.7 ± 0.01	131.7 ± 0.01	111.7 ± 0.01
	Rh	187.9 ± 0.01	188.6 ± 0.01	187.9 ± 0.01	154.2 ± 0.01	147.3 ± 0.01	108.6 ± 0.01
red	CS	135.9 ± 0.01	147.1 ± 0.01	209.3 ± 0.01	164.3 ± 0.01	128.2 ± 0.01	113.7 ± 0.01
	65 °C	127.6 ± 0.01	134.8 ± 0.01	129.8 ± 0.01	131.5 ± 0.01	123.7 ± 0.01	105.4 ± 0.01
	75 °C	159.3 ± 0.01	173.2 ± 0.01	174.3 ± 0.01	161.5 ± 0.01	134.3 ± 0.01	138.7 ± 0.01
	Le	203.2 ± 0.01	197.1 ± 0.01	198.2 ± 0.01	184.3 ± 0.01	122.1 ± 0.01	101.5 ± 0.01
	Li	207.6 ± 0.01	215.4 ± 0.01	165.4 ± 0.01	163.2 ± 0.01	99.3 ± 0.01	72.1 ± 0.01
	Rh	172.6 ± 0.01	182.1 ± 0.01	174.3 ± 0.01	169.8 ± 0.01	123.2 ± 0.01	88.7 ± 0.01

Data are expressed as the mean, *n* = 6, and standard deviation (±SD).

**Table 12:** The color of yoghurts with added lyophilized anthocyanin pigments obtained from juices of potato varieties with red or purple flesh (0.4 g/300 mL).

Flesh Color	Variety	Variant	0 Day			2 Day			7 Day		
			L*	a*	b*	L*	a*	b*	L*	a*	b*
		yoghurt	81.6 ^f A^	−2.12 ^aA^	5.47 ^jA^	81.3 ^gA^	−2.17 ^aA^	5.41 ^hA^	82.1 ^gA^	−2.38 ^Aa^	5.44 ^gA^
purple	Double Fun	CS	68.0 ^aA^	8.53 ^hA^	−3.32 ^cA^	68.6 ^aA^	8.12 ^gA^	−2.71 ^cB^	69.5 ^aB^	8.13 ^fA^	−2.54 ^bB^
		65 °C	68.7 ^aA^	6.81 ^fA^	−1.21 ^eA^	69.7 ^bB^	6.81 ^eA^	−1.04 ^dA^	70.6 ^bC^	6.72 ^dA^	−0.81 ^cA^
		75 °C	69.1 ^bAB^	6.62 ^fA^	−0.68 ^fA^	69.5 ^bB^	6.44 ^eA^	−0.41 ^eA^	68.9 ^aA^	6.15 ^Da^	−0.05 ^cA^
		Le	69.5 ^aB^	9.51 ^iB^	−4.60 ^bA^	68.5 ^aA^	9.53 ^hB^	−4.90 ^aA^	70.2 ^bC^	8.32 ^Fa^	−3.28 ^aB^
		Li	68.2 ^aA^	9.90 ^iB^	−5.11 ^aA^	69.4 ^bB^	8.64 ^gA^	−3.93 ^bB^	69.6 ^aB^	8.85 ^fA^	−3.88 ^aB^
		Rh	69.9 ^bA^	8.08 ^hA^	−2.81 ^dA^	69.8 ^bA^	7.99 ^fA^	−2.74 ^cA^	70.2 ^bA^	7.77 ^Ea^	−2.18 ^bA^
	Provita	CS	73.8 ^bA^	2.55 ^bA^	5.31 ^jA^	73.9 ^cA^	2.55 ^bA^	5.77 ^hAB^	74.8 ^dB^	2.48 ^Ba^	6.01 ^hB^
		65 °C	74.5 ^cB^	2.78 ^bA^	6.32 ^kA^	73.9 ^cA^	2.77 ^bA^	6.26 ^iA^	73.7 ^cA^	2.72 ^bA^	6.15 ^hA^
		75 °C	74.5 ^cA^	2.62 ^bA^	6.09 ^kA^	74.5 ^dA^	2.78 ^bA^	6.30 ^iA^	73.9 ^cA^	2.65 ^bA^	6.06 ^hA^
		Le	76.8 ^eA^	2.83 ^bA^	4.94 ^iA^	76.5 ^fA^	2.80 ^bA^	5.23 ^hA^	76.5 ^fA^	2.94 ^Ba^	5.18 ^gA^
		Li	76.3 ^eA^	2.74 ^bA^	5.85 ^jA^	75.8 ^eA^	2.70 ^bA^	6.06 ^iA^	75.9 ^eA^	2.88 ^Ba^	5.90 ^gA^
		Rh	76.4 ^eB^	2.74 ^bA^	5.92 ^jA^	74.2 ^dA^	3.09 ^cB^	5.64 ^hA^	74.6 ^dA^	3.19 ^cB^	5.57 ^gA^
red	Lily Rose	CS	74.7 ^cA^	2.72 ^bA^	6.87 ^kA^	74.5 ^dA^	2.59 ^bA^	6.91 ^iA^	74.2 ^dA^	2.42 ^bA^	6.79 ^hA^
		65 °C	74.6 ^cA^	3.45 ^cA^	5.82 ^jA^	74.8 ^dA^	3.26 ^cA^	5.94 ^hA^	75.9 ^eB^	3.18 ^Ca^	6.15 ^hA^
		75 °C	74.2 ^cA^	4.02 ^dA^	5.37 ^jA^	74.6 ^dA^	3.95 ^cA^	5.48 ^hA^	73.8 ^cA^	3.78 ^Ca^	5.33 ^gA^
		Le	75.5 ^dB^	7.65 ^gB^	1.93 ^gA^	74.3 ^dA^	7.80 ^fB^	2.27 ^Fb^	75.1 ^eB^	6.39 ^dA^	3.19 ^eC^
		Li	75.9 ^eB^	6.73 ^fB^	2.42 ^hA^	75.2 ^eB^	5.55 ^dA^	3.99 ^gB^	74.2 ^dA^	8.10 ^Fc^	2.01 ^dA^
		Rh	75.0 ^dB^	5.71 ^eA^	4.76 ^iA^	73.7 ^cA^	5.69 ^dA^	5.07 ^hA^	74.4 ^dAB^	6.09 ^Da^	4.76 ^fA^

Data are expressed as the mean. *n* = 2. Results in the same column followed by different letters indicate significant differences according to Duncan’s test at *p* < 0.05 between different variants in varieties (small letters); results in the same line followed by different letters indicate significant differences according to Duncan’s test at *p* < 0.05 between days (big letters), as determined by one-way ANOVA.

**Table 13:** Lactic acid bacteria counts (log10 CFU/mL) and pH in yoghurts without or with addition of lyophilized potato pigments during 7 days of storage (Lab-MRS-5 estimated counts of lactobacilli in MRS agar at pH 5.2, Lac-MRS-17 estimated counts of lactococci on M17 agar).

Flesh Colour	Variant	2 Days		7 Days		0 Days	2 Days	7 Day
		Lab-MRS-5	Lac-M17-7	Lab-MRS-5	Lac-M17-7	pH		
	yoghurt	7.67	7.93	7.56	8.32	4.10	4.30	4.15
purple	CS	7.54	8.04	7.65	8.79	4.12	4.29	4.20
	65 °C	7.75	8.45	7.71	8.67	3.98	4.15	4.07
	75 °C	7.50	7.71	7.59	8.72	3.92	4.07	4.16
	Le	7.39	8.79	7.72	9.38	4.35	4.17	4.38
	Li	8.05	9.82	7.95	9.19	4.38	4.33	4.34
	Rh	7.98	9.49	7.95	9.49	4.38	4.36	4.34
red	CS	7.62	9.85	7.71	8.57	4.01	4.11	4.14
	65 °C	7.50	9.49	7.73	9.72	4.10	4.28	4.19
	75 °C	7.56	9.74	7.57	9.58	4.02	4.12	4.23
	Le	7.84	9.25	7.55	8.91	4.53	4.39	4.34
	Li	7.79	8.36	7.73	8.70	4.52	4.37	4.34
	Rh	7.85	9.56	7.60	9.32	4.53	4.37	4.17

Data are expressed as the mean, *n* = 2.

## Data Availability

Data will be made available upon request.

## Supplementary Materials

The supporting information can be downloaded at: https://www.mdpi.com/article/10.3390/ijms252011116/s1.
