# Supplementary material for: Studying the Stability of Anthocyanin Pigments Isolated from Juices of Colored-Fleshed Potatoes
Source: Int J Mol Sci. 2024 Oct 16;25(20):11116. doi: 10.3390/ijms252011116 (PMC11507568; doi:10.3390/ijms252011116)
Supplement: Supplementary file 1 [file ijms-25-11116-s001.zip › ijms-3249271-supplementary.pdf]

Table S1. Total polyphenol content (TP) (mg GAE/1 g d.m) and antioxidant activity measured by the ABTS (umolT/1g) method in fruit and vegetable concentrates.

**Concentrate**	**TP**	**ABTS+**
Lemon	3.36[a]	43.78[a]
Lime	4.88[b]	49.10[ab]
Rhubarb	27.5[d]	454.1[d]
Apple	9.18[c]	95.72[c]
Grapefruit	4.86[b]	51.14[b]

Data are expressed as the mean, n=6. Results in the same column followed by different letters indicate significant differences according to Duncan's test at $p < 0.05$ between different concentrates as determined and by one-way ANOVA

Table S2. Model study of color change L* parameter under the influence of temperature from 60°C to 100 °C after 5, 10, 15 minutes in pigment extracts of purple or red potato juices: non-pasteurized and without additives (control sample CS); after pasteurization in temperature 65°C and 75°C; with additives of lemon (Le), lime (Li) and rhubarb (Rh) concentrates.

Flesh color	Variant	Non-pasteurized			60°C			70°C			80°C			90°C			100°C		
		5	10	15	5	10	15	5	10	15	5	10	15	5	10	15	5	10	15
purple	CS	5.30^{bA}	5.53^{bA}	5.64^{bA}	21.2^{bB}	25.0^{bC}	28.4^{bD}	29.9^{cE}	34.1^{cG}	34.7^{cG}	31.5^{cF}	35.7^{cH}	35.9^{cH}	38.9^{cJ}	38.3^{bJ}	38.0^{cJ}	39.2^{cK}	38.7^{cJ}	37.0^{cI}
	65°C	20.9^{eA}	21.3^{eA}	20.9^{eA}	32.1^{cB}	34.7^{dC}	35.9^{cD}	36.0^{dE}	36.0^{dE}	36.2^{dE}	36.1^{dE}	37.4^{dF}	37.2^{dF}	38.6^{cG}	38.8^{bG}	39.0^{dH}	39.2^{cH}	38.3^{cG}	37.3^{cF}
	75°C	1.80^{aA}	1.64^{aA}	1.90^{aA}	8.20^{aB}	13.5^{aC}	14.7^{aD}	21.1^{aE}	26.7^{aF}	29.8^{aG}	26.3^{aF}	30.5^{aH}	31.1^{aI}	31.9^{aI}	34.5^{aK}	34.1^{aK}	33.4^{aJ}	34.7^{aK}	34.0^{aK}
	Le	10.9^{cA}	10.8^{cA}	10.7^{cA}	38.4^{eB}	38.9^{eB}	38.3^{dB}	40.6^{eD}	41.2^{eE}	39.4^{eC}	39.7^{eC}	41.8^{eE}	39.7^{eC}	43.6^{dG}	41.8^{cE}	40.0^{eC}	44.1^{dG}	42.4^{dF}	41.6^{dE}
	Li	14.2^{dA}	14.4^{dA}	14.3^{dA}	36.6^{dB}	40.2^{fE}	37.8^{dC}	42.1^{fG}	41.6^{eF}	39.3^{eD}	41.4^{fF}	41.7^{eF}	40.5^{fE}	44.4^{eI}	44.3^{dI}	42.3^{fG}	43.8^{dH}	43.9^{eH}	42.5^{eG}
	Rh	5.41^{bA}	5.18^{bA}	5.30^{bA}	21.6^{bB}	26.1^{cC}	27.7^{bD}	28.6^{bE}	31.6^{bG}	33.7^{bI}	30.4^{bF}	32.7^{bH}	32.4^{bH}	35.5^{bJ}	$38.8^{bŁ}$	36.8^{bK}	37.9^{bL}	37.9^{bL}	36.2^{bK}
red	CS	69.9^{hF}	70.5^{iG}	70.1^{iG}	65.3^{gE}	64.7^{iD}	63.7^{fC}	65.1^{hE}	63.7^{gC}	62.9^{gB}	64.2^{iD}	63.7^{hC}	61.8^{iA}	63.3^{gC}	62.9^{fB}	63.6^{iC}	63.8^{fC}	64.6^{hD}	62.2^{gB}
	65°C	68.4^{hF}	68.4^{hF}	68.4^{hF}	65.3^{gE}	65.1^{iE}	64.2^{fgD}	64.9^{hD}	64.2^{gD}	64.2^{hD}	64.1^{iD}	62.7^{gB}	62.6^{jB}	63.5^{gC}	62.9^{fB}	61.3^{hA}	63.4^{fC}	63.4^{gC}	62.8^{gB}
	75°C	65.6^{gH}	65.6^{gH}	65.7^{gH}	62.6^{fG}	61.5^{gF}	60.9^{eE}	60.5^{gE}	59.3^{fD}	59.1^{fD}	59.6^{gD}	58.5^{fC}	57.8^{gB}	59.1^{fD}	56.6^{eA}	56.9^{gA}	59.6^{eD}	58.8^{fC}	57.6^{fB}
	Le	70.7^{iJ}	68.9^{hH}	68.9^{hH}	67.6^{hG}	66.2^{jF}	65.0^{gE}	75.3^{jL}	69.3^{hI}	71.1^{jK}	62.7^{hC}	62.7^{gC}	60.0^{hB}	63.7^{gD}	63.9^{gD}	59.9^{hA}	67.9^{gG}	67.7^{iG}	66.2^{hF}
	Li	77.7^{jG}	77.7^{jG}	77.7^{jG}	74.9^{iD}	75.5^{kE}	71.0^{hA}	75.7^{jE}	75.0^{iE}	76.5^{kF}	76.5^{kF}	74.4^{jD}	72.9^{lB}	75.5^{iE}	73.3^{iC}	74.1^{kD}	75.2^{iE}	75.2^{jE}	73.6^{jC}
	Rh	62.9^{fB}	62.9^{fB}	63.3^{fC}	68.0^{hF}	65.1^{iD}	61.4^{eA}	69.7^{iG}	68.9^{hF}	67.4^{iE}	69.1^{jG}	68.3^{iF}	67.5^{kE}	68.9^{hF}	67.7^{hE}	67.2^{jE}	69.1^{hG}	68.2^{iF}	67.6^{iE}

Data are expressed as the mean, n=6. Results in the same column followed by different letters indicat significant differences according to Duncan's test at $p < 0.05$ between different flesh colors and varieties (small letters); between different temperatures (big letters) as determined and by one-way ANOVA

Table S3. Model study content of total polyphenol (TP) (mg GAE/1 g d.m) as affected by temperature changes from 60°C to 100 °C in pigment extracts of purple or red potato juices: non-pasteurized and without additives (control sample CS); after pasteurization in temperature 65°C and 75°C; with additives of lemon (Le), lime (Li) and rhubarb (Rh) concentrates.

Flesh color	Variant	Non-pasteurized			60°C			70°C			80°C			90°C			100°C		
		**5**	**10**	**15**	**5**	**10**	**15**	**5**	**10**	**15**	**5**	**10**	**15**	**5**	**10**	**15**	**5**	**10**	**15**
purple	CS	147.1 ±0.01	151.6 ±0.01	151.6 ±0.01	150.8 ±0.01	161.6 ±0.01	124.6 ±0.01	163.0 ±0.01	158.0 ±0.01	162.1 ±0.01	143.8 ±0.15	165.8 ±0.15	162.4 ±0.15	159.4 ±0.15	148.3 ±0.15	135.8 ±0.15	156.9 ±0.15	161.3 ±0.15	153.8 ±0.15
	65°C	163.8 ±0.01	154.6 ±0.01	155.5 ±0.01	149.9 ±0.01	162.4 ±0.01	152.4 ±0.01	151.6 ±0.15	129.9 ±0.15	153.3 ±0.15	159.6 ±0.15	146.0 ±0.15	113.8 ±0.15	131.3 ±0.15	158.3 ±0.15	150.2 ±0.15	129.6 ±0.15	162.4 ±0.15	161.6 ±0.15
	75°C	166.6 ±0.01	162.1 ±0.01	173.8 ±0.01	162.1 ±0.01	163.8 ±0.01	172.4 ±0.01	162.4 ±0.01	153.8 ±0.01	168.8 ±0.01	162.1 ±0.15	151.9 ±0.15	170.5 ±0.15	158.8 ±0.15	152.7 ±0.15	163.3 ±0.15	148.5 ±0.15	163.3 ±0.15	159.9 ±0.15
	Le	155.4 ±0.01	153.3 ±0.01	154.7 ±0.01	144.9 ±0.05	143.5 ±0.05	142.9 ±0.05	141.5 ±0.05	141.5 ±0.05	146.3 ±0.05	141.5 ±0.05	144.9 ±0.05	144.2 ±0.05	139.4 ±0.01	143.5 ±0.01	145.6 ±0.01	115.8 ±0.01	151.9 ±0.01	141.5 ±0.01
	Li	125.5 ±0.01	122.7 ±0.01	142.9 ±0.01	106.0 ±0.05	123.4 ±0.05	133.8 ±0.05	82.4 ±0.05	112.3 ±0.05	138.7 ±0.05	138.7 ±0.05	126.2 ±0.05	133.1 ±0.05	138.7 ±0.01	131.0 ±0.01	136.6 ±0.01	145.6 ±0.01	143.5 ±0.01	119.2 ±0.01
	Rh	158.8 ±0.01	153.3 ±0.01	144.2 ±0.01	149.1 ±0.01	140.1 ±0.01	133.8 ±0.01	81.7 ±0.05	142.9 ±0.05	109.5 ±0.05	141.5 ±0.05	146.3 ±0.05	152.6 ±0.05	133.1 ±0.05	149.8 ±0.05	142.9 ±0.05	158.1 ±0.01	144.9 ±0.01	140.1 ±0.01
red	CS	115.5 ±0.01	109.9 ±0.01	99.9 ±0.01	99.6 ±0.01	105.5 ±0.01	95.5 ±0.01	100.5 ±0.01	99.6 ±0.01	104.4 ±0.01	102.4 ±0.01	99.6 ±0.01	32.4 ±0.01	103.3 ±0.01	99.9 ±0.01	99.1 ±0.01	99.6 ±0.01	104.9 ±0.01	102.4 ±0.01
	65°C	98.0 ±0.01	96.3 ±0.01	96.9 ±0.01	123.5 ±0.01	102.4 ±0.01	98.8 ±0.01	99.1 ±0.01	101.9 ±0.01	97.4 ±0.01	96.0 ±0.01	99.6 ±0.01	96.9 ±0.01	96.9 ±0.01	101.0 ±0.01	94.9 ±0.01	95.5 ±0.01	100.5 ±0.01	101.3 ±0.01
	75°C	114.9 ±0.01	116.6 ±0.01	115.5 ±0.01	102.1 ±0.01	113.3 ±0.01	112.7 ±0.01	108.0 ±0.01	111.6 ±0.01	109.9 ±0.01	116.9 ±0.01	118.5 ±0.01	114.6 ±0.01	112.4 ±0.01	113.0 ±0.01	103.0 ±0.01	98.8 ±0.01	115.5 ±0.01	114.6 ±0.1
	Le	59.4 ±0.15	52.4 ±0.15	58.8 ±0.15	108.0 ±0.15	192.2 ±0.15	122.1 ±0.15	145.8 ±0.05	117.7 ±0.05	160.2 ±0.05	114.6 ±0.01	64.6 ±0.01	100.2 ±0.01	147.1 ±0.01	138.5 ±0.01	149.4 ±0.01	56.3 ±0.01	98.8 ±0.01	160.8 ±0.01
	Li	92.1 ±0.15	136.3 ±0.15	104.1 ±0.15	140.2 ±0.15	124.1 ±0.15	90.8 ±0.15	141.6 ±0.15	124.4 ±0.15	131.6 ±0.15	121.0 ±0.01	99.1 ±0.01	122.4 ±0.01	139.9 ±0.01	134.4 ±0.01	116.9 ±0.01	76.6 ±0.01	132.4 ±0.01	129.4 ±0.01
	Rh	129.4 ±0.01	165.8 ±0.01	142.7 ±0.01	129.4 ±0.05	157.4 ±0.05	148.5 ±0.05	125.2 ±0.05	133.8 ±0.05	106.6 ±0.05	150.5 ±0.01	111.9 ±0.01	136.0 ±0.01	104.6 ±0.01	101.6 ±0.01	114.4 ±0.01	161.0 ±0.01	153.5 ±0.01	124.6 ±0.01

Data are expressed as the mean and standard deviation (±SD), n=6.

Table S4. Model study antioxidative activity measured by the ABTS+ method (umolT/1g) as affected by temperature changes from 60°C to 100 °C after 5, 10, 15 minutes in pigment extracts of purple or red potato juices: non-pasteurized and without additives (control sample CS); after pasteurization in temperature 65°C and 75°C; with additives of lemon (Le), lime (Li) and rhubarb (Rh) concentrates.

Flesh colour	Variant	Non-pasteurized			60°C			70°C			80°C			90°C			100°C		
		5	10	15	5	10	15	5	10	15	5	10	15	5	10	15	5	10	15
purple	CS	1967.8	1963.7	1951.4	1971.9	2008.7	1881.9	1914.6	1939.1	1779.6	1984.1	1906.4	1963.7	1738.7	2012.8	1943.2	1959.6	1984.1	1931.0
	65°C	1869.6	2053.7	2016.9	1943.2	1914.6	1922.8	1914.6	1767.3	1984.1	1939.1	1665.0	1661.0	1738.7	1943.2	1902.3	1726.4	1832.8	1943.2
	75°C	2094.6	2082.3	2106.9	2176.4	2090.5	2098.7	2156.0	2102.8	2098.7	1926.9	2131.4	2061.9	1988.2	2029.1	1992.3	1763.2	1329.6	1861.4
	Le	511.4	1769.4	1861.4	20.5	40.9	225.0	51.1	1841.0	204.6	194.3	40.9	20.5	2280.7	2055.7	71.6	1963.7	675.0	2106.9
	Li	214.8	1902.3	30.7	1984.1	900.0	112.5	378.4	1851.2	777.3	2137.6	1994.4	2209.1	2025.1	1963.7	409.1	2055.7	2168.2	2884.2
	Rh	2250.1	2198.9	951.2	2168.2	981.8	2229.6	2025.1	276.1	2567.1	306.8	2291.0	2025.1	1943.2	2066.0	2260.3	2239.8	122.7	2331.9
red	CS	1746.9	1791.9	1640.5	1648.7	1746.9	1718.2	1726.4	1579.1	1697.8	1861.4	1661.0	1681.4	1648.7	1796.0	1677.3	1673.2	1767.3	1697.8
	65°C	1550.5	1575.0	1607.8	1538.2	1423.7	1726.4	1566.9	1611.9	1603.7	1566.9	1521.9	1546.4	1624.1	1595.5	1534.1	1558.7	1587.3	1566.9
	75°C	1832.8	1779.6	1832.8	1742.8	1910.5	1779.6	1804.1	1701.9	1853.2	1881.9	1824.6	1800.0	1841.0	1681.4	1665.0	1591.4	1718.2	1701.9
	Le	1080.0	1165.9	1010.5	1800.0	1554.6	1456.4	1644.6	1579.1	1697.8	1775.5	1685.5	1800.0	1771.4	1575.0	1620.0	1096.4	1276.4	1808.2
	Li	1067.8	1395.0	1489.1	1525.9	1566.9	1570.9	1460.5	1489.1	1272.3	1460.5	1341.9	1591.4	1534.1	1292.8	1579.1	1603.7	1591.4	1620.0
	Rh	1616.0	1894.1	1849.1	1677.3	1693.7	1665.0	1783.7	1791.9	1783.7	1836.9	1804.1	1755.0	1816.4	1620.0	1640.5	1853.2	1804.1	1951.4

Table S5. Model study antioxidative activity measured by the DPPH method (umolT/1g) as affected by temperature changes from 60°C to 100 °C after 5, 10, 15 minutes in pigment extracts of purple or red potato juices: non-pasteurized and without additives (control sample CS); after pasteurization in temperature 65°C and 75°C; with additives of lemon (Le), lime (Li) and rhubarb (Rh) concentrates.

Flesh colour	Variant	Non-pasteurized			60°C			70°C			80°C			90°C			100°C		
		5	10	15	5	10	15	5	10	15	5	10	15	5	10	15	5	10	15
purple	CS	114.7	123.2	122.7	128.0	123.7	150.8	129.0	141.3	133.8	130.1	148.2	130.6	134.3	146.6	138.1	137.5	148.2	141.8
	65°C	118.4	123.7	121.6	122.7	135.9	146.0	117.9	122.1	125.3	114.7	122.7	132.8	123.7	106.2	122.7	132.2	104.6	124.8
	75°C	103.0	105.1	104.1	122.7	130.6	124.8	122.1	131.2	124.3	121.1	132.2	125.3	128.0	115.2	122.7	132.8	110.5	120.0
	Le	323.9	355.8	325.2	334.5	288.1	359.8	326.6	310.6	327.9	248.2	316.0	281.4	347.8	367.7	305.3	297.4	327.9	349.1
	Li	334.5	353.1	335.9	370.4	329.2	373.0	345.2	322.6	346.5	334.5	366.4	357.1	349.1	371.7	309.3	312.0	341.2	362.4
	Rh	302.7	325.2	306.7	306.7	285.4	309.3	292.1	280.1	293.4	309.3	304.0	309.3	349.1	358.4	346.5	373.0	375.7	398.3
red	CS	150.3	152.9	151.9	151.9	150.3	148.2	153.5	152.9	150.3	142.8	150.3	153.5	146.6	146.0	165.1	152.4	150.3	155.6
	65°C	152.9	150.8	151.3	140.2	143.4	147.1	149.2	145.0	149.2	147.1	146.0	149.7	147.1	144.4	149.2	147.6	143.4	149.7
	75°C	154.0	144.4	143.4	158.8	143.9	143.4	160.9	146.6	145.0	145.0	144.4	141.8	143.9	143.4	141.3	143.9	141.8	140.2
	Le	57.9	114.7	113.6	149.7	156.1	155.6	137.0	155.1	140.2	139.7	123.7	139.1	154.5	154.5	71.7	160.9	157.2	163.6
	Li	135.4	137.5	128.5	152.9	70.1	139.1	128.5	135.4	140.7	142.8	128.5	131.7	133.8	136.5	103.0	132.2	124.8	181.1
	Rh	118.9	123.2	132.2	131.2	145.0	124.8	129.6	138.1	120.5	148.2	133.3	114.7	127.4	133.8	150.8	141.8	127.4	138.1

Table S6. Model study antioxidative activity measured by the FRAP method (umolT/1g) as affected by temperature changes from 60°C to 100 °C after 5, 10, 15 minutes in pigment extracts of purple or red potato juices: non-pasteurized and without additives (control sample CS); after pasteurization in temperature 65°C and 75°C; with additives of lemon (Le), lime (Li) and rhubarb (Rh) concentrates.

Flesh colour	Variant	Non-pasteurized			60°C			70°C			80°C			90°C			100°C		
		5	10	15	5	10	15	5	10	15	5	10	15	5	10	15	5	10	15
purple	CS	414.9	399.8	400.7	391.3	398.9	350.9	389.5	367.8	381.0	387.6	355.6	355.6	380.0	358.4	373.4	374.4	355.6	366.9
	65°C	408.3	398.9	402.6	400.7	377.2	359.3	409.2	401.7	396.0	414.9	400.7	382.9	398.9	429.9	400.7	383.8	432.8	397.0
	75°C	435.6	431.8	433.7	494.9	480.8	480.8	495.8	479.8	500.5	497.7	477.9	481.7	485.5	508.1	485.5	477.0	516.5	499.6
	Le	1206.6	1150.1	1204.2	1187.8	1270.1	1143.1	1201.9	1251.3	1199.5	1340.7	1267.8	1281.9	1164.2	1128.9	1239.5	1227.8	1199.5	1161.9
	Li	1187.8	1154.8	1185.4	1124.2	1197.2	1119.5	1168.9	1208.9	1166.6	1187.8	1131.3	1147.8	1161.9	1121.9	1274.8	1227.8	1176.0	1138.3
	Rh	1244.2	1204.2	1213.7	1213.7	1274.8	1232.5	1263.1	1284.2	1260.7	1232.5	1241.9	1232.5	1161.9	1145.4	1166.6	1119.5	1114.8	1074.8
red	CS	445.9	441.2	443.1	443.1	445.9	449.7	443.1	441.2	445.9	459.1	445.9	440.3	452.5	453.5	419.6	442.2	445.9	436.5
	65°C	441.2	445.0	444.0	463.8	458.2	451.6	447.8	455.3	447.8	451.6	453.5	446.9	451.6	456.3	447.8	450.6	458.2	446.9
	75°C	439.3	456.3	458.2	430.9	457.2	458.2	427.1	452.5	455.3	455.3	456.3	461.0	457.2	458.2	461.9	457.2	461.0	463.8
	Le	609.7	509.0	510.9	446.9	435.6	436.5	469.5	437.5	463.8	464.8	509.9	465.7	438.4	438.4	585.2	427.1	433.7	422.4
	Li	472.3	468.5	484.5	441.2	588.1	465.7	484.5	472.3	462.9	459.1	484.5	478.9	475.1	470.4	529.7	477.9	491.1	391.3
	Rh	491.1	493.9	477.9	479.8	455.3	491.1	482.6	467.6	498.6	449.7	476.1	509.0	486.4	475.1	445.0	461.0	486.4	467.6

Table S7. Model study antioxidative activity measured by the ABTS+ (umolT/1g), DPPH (umolT/1g), and FRAP (umolT/1g) methods as affected by pH changes from 1 to 11 in pigment extracts of purple or red potato juices: non-pasteurized and without additives (control sample CS); after pasteurization in temperature 65°C and 75°C; with additives of lemon (Le), lime (Li) and rhubarb (Rh) concentrates.

Flesh color	Variant	ABTS+						DPPH						FRAP					
		1	3	5	7	9	11	1	3	5	7	9	11	1	3	5	7	9	11
purple	CS	2411.6	2770.6	2807.5	2816.7	2641.8	2402.4	968.9	381.1	340.5	327.4	204.3	121.9	1134.6	1600.6	1001.2	810.6	774.6	541.6
	65°C	2153.9	2549.7	2669.4	2568.1	2227.6	2292.0	967.8	255.7	299.9	295.1	204.3	142.2	1361.3	1621.8	1100.8	1071.1	817.0	562.8
	75°C	2006.6	2558.9	2614.2	2733.8	2402.4	2227.6	964.2	346.5	358.4	287.9	149.3	154.1	1200.3	1799.7	1179.1	965.2	838.1	584.0
	Le	1831.8	2384.0	2246.0	1933.0	1841.0	1887.0	918.8	357.2	264.0	266.4	142.2	70.5	969.4	1596.4	1134.6	918.6	808.5	721.6
	Li	1850.2	1969.8	2181.5	1868.6	1979.0	1776.5	909.2	442.1	291.5	195.9	136.2	47.8	986.4	1600.6	1134.6	922.9	821.2	658.1
	Rh	1555.6	2439.3	2117.1	1997.4	2080.3	1785.7	831.6	394.3	261.7	210.3	123.1	19.1	903.8	1568.8	1230.0	1024.5	922.9	711.1
red	CS	1685.5	2241.9	3060.1	2323.7	2070.1	1996.4	773.1	836.9	265.5	289.9	168.9	35.1	1409.6	1467.9	1230.7	937.0	707.4	613.2
	65°C	1530.0	2102.8	2225.5	2291.0	2021.0	1832.8	786.9	328.2	297.4	265.5	157.2	35.1	1402.0	1595.9	1253.3	1085.8	876.8	711.1
	75°C	1906.4	2233.7	2634.6	2405.5	2266.4	2045.5	800.8	340.9	351.5	262.3	145.5	24.4	1366.3	1460.4	1403.9	1083.9	933.3	820.3
	Le	1824.6	2307.3	2307.3	2438.2	1783.7	1783.7	822.0	871.9	484.3	292.1	130.6	68.0	1420.9	1516.9	1304.1	1210.0	905.0	566.2
	Li	2217.3	2601.9	2160.1	2160.1	1611.9	1579.1	848.5	568.2	296.3	300.6	107.3	107.3	1424.6	1243.9	1426.5	1083.9	886.2	605.7
	Rh	1652.8	2176.4	2348.2	2274.6	1808.2	1726.4	832.6	809.2	315.4	257.0	121.1	107.3	1394.5	1426.5	1210.0	969.0	833.5	665.9

Table S8. The value of the L* parameter every 15 sec for 6 min of the yoghurts immediately after adding the lyphilized pigments of purple or red potato juices: non-pasteurized and without additives (CS); after pasteurization in temperature 65°C and 75°C; with additives of lemon (Le), lime (Li) and rhubarb (Rh) concentrates.

Time [sec]	yoghurt	purple						red					
		CS	65°C	75°C	Le	Li	Rh	CS	65°C	75°C	Le	Li	Rh
00:00	81.7	67.3	68.6	69.0	67.8	64.2	68.5	74.8	74.9	74.4	75.4	76.0	75.0
00:15	81.7	67.6	68.9	69.0	68.2	65.1	68.9	74.5	74.3	73.9	75.4	76.0	75.1
00:30	81.7	67.4	68.4	69.2	68.5	65.5	69.0	74.8	74.4	73.9	75.5	75.9	75.0
00:45	81.7	67.8	68.8	69.0	68.8	66.2	69.2	74.8	74.7	73.9	75.5	76.0	75.0
01:00	81.7	67.7	68.6	69.2	69.0	66.8	69.5	74.5	74.4	74.0	75.5	76.0	75.1
01:15	81.7	68.0	68.7	69.3	69.2	67.3	69.7	75.1	74.9	74.1	75.5	76.0	75.0
01:30	81.7	67.9	68.3	69.0	69.3	67.8	69.8	73.7	74.4	74.1	75.5	76.1	74.8
01:45	81.7	68.0	68.7	69.1	69.5	68.0	70.0	74.6	74.5	74.2	75.4	76.0	75.0
02:00	81.7	68.1	68.7	68.9	69.6	68.4	69.9	75.0	74.8	74.4	75.4	75.9	75.1
02:15	81.7	68.1	68.9	69.2	69.7	68.7	70.1	74.1	74.7	74.5	75.5	76.0	75.1
02:30	81.7	68.3	69.0	69.1	69.7	69.0	70.0	75.0	74.6	73.8	75.6	75.9	75.0
02:45	81.7	67.9	68.5	69.0	69.9	69.2	70.2	74.9	74.4	74.1	75.5	76.0	75.1
03:00	81.7	68.3	69.1	69.2	69.9	69.3	70.4	74.8	74.5	74.4	75.4	76.0	75.1
03:30	81.7	68.3	68.5	69.0	70.0	69.8	70.5	74.9	74.6	74.1	75.6	76.0	74.9
04:00	81.7	68.4	68.8	69.1	70.2	69.9	70.5	74.8	74.7	74.0	75.5	75.9	75.0
04:30	81.7	68.3	69.0	69.2	70.2	70.1	70.7	74.9	74.6	74.4	75.4	75.9	75.0
05:00	81.7	68.4	68.3	69.1	70.3	70.3	70.6	75.0	74.6	74.0	75.5	76.0	74.8
05:30	81.0	68.4	68.9	69.0	70.2	70.3	70.7	74.4	74.6	74.3	75.6	76.0	75.0
06:00	80.9	68.3	68.8	69.1	70.2	70.3	70.6	74.8	74.6	74.5	75.5	75.9	75.0
